# A Near-Complete Haplotype-Phased Genome of the Dikaryotic Wheat Stripe Rust Fungus *Puccinia striiformis* f. sp. *tritici* Reveals High Interhaplotype Diversity

**DOI:** 10.1128/mBio.02275-17

**Published:** 2018-02-20

**Authors:** Benjamin Schwessinger, Jana Sperschneider, William S. Cuddy, Diana P. Garnica, Marisa E. Miller, Jennifer M. Taylor, Peter N. Dodds, Melania Figueroa, Robert F. Park, John P. Rathjen

**Affiliations:** a Research School of Biology, the Australian National University, Acton, ACT, Australia; b Centre for Environment and Life Sciences, CSIRO Agriculture and Food, Perth, WA, Australia; c Plant Breeding Institute, Faculty of Agriculture and Environment, the University of Sydney, Narellan, NSW, Australia; d NSW Department of Primary Industries, Elizabeth Macarthur Agricultural Institute, Menangle, NSW, Australia; e Department of Plant Pathology, University of Minnesota, St. Paul, Minnesota, USA; f Stakman-Borlaug Center for Sustainable Plant Health, University of Minnesota, St. Paul, Minnesota, USA; g Black Mountain Laboratories, CSIRO Agriculture and Food, Canberra, ACT, Australia; University of Córdoba

**Keywords:** Dikaryon, basidiomycetes, genomics, plant pathogens

## Abstract

A long-standing biological question is how evolution has shaped the genomic architecture of dikaryotic fungi. To answer this, high-quality genomic resources that enable haplotype comparisons are essential. Short-read genome assemblies for dikaryotic fungi are highly fragmented and lack haplotype-specific information due to the high heterozygosity and repeat content of these genomes. Here, we present a diploid-aware assembly of the wheat stripe rust fungus *Puccinia striiformis* f. sp. *tritici* based on long reads using the FALCON-Unzip assembler. Transcriptome sequencing data sets were used to infer high-quality gene models and identify virulence genes involved in plant infection referred to as effectors. This represents the most complete *Puccinia striiformis* f. sp. *tritici* genome assembly to date (83 Mb, 156 contigs, *N*_50_ of 1.5 Mb) and provides phased haplotype information for over 92% of the genome. Comparisons of the phase blocks revealed high interhaplotype diversity of over 6%. More than 25% of all genes lack a clear allelic counterpart. When we investigated genome features that potentially promote the rapid evolution of virulence, we found that candidate effector genes are spatially associated with conserved genes commonly found in basidiomycetes. Yet, candidate effectors that lack an allelic counterpart are more distant from conserved genes than allelic candidate effectors and are less likely to be evolutionarily conserved within the *P. striiformis* species complex and *Pucciniales*. In summary, this haplotype-phased assembly enabled us to discover novel genome features of a dikaryotic plant-pathogenic fungus previously hidden in collapsed and fragmented genome assemblies.

## IMPORTANCE

Current representations of eukaryotic microbial genomes are haploid, hiding the genomic diversity intrinsic to diploid and polyploid life forms. This hidden diversity contributes to the organism’s evolutionary potential and ability to adapt to stress conditions. Yet, it is challenging to provide haplotype-specific information at a whole-genome level. Here, we take advantage of long-read DNA sequencing technology and a tailored-assembly algorithm to disentangle the two haploid genomes of a dikaryotic pathogenic wheat rust fungus. The two genomes display high levels of nucleotide and structural variations, which lead to allelic variation and the presence of genes lacking allelic counterparts. Nonallelic candidate effector genes, which likely encode important pathogenicity factors, display distinct genome localization patterns and are less likely to be evolutionary conserved than those which are present as allelic pairs. This genomic diversity may promote rapid host adaptation and/or be related to the age of the sequenced isolate since last meiosis.

## INTRODUCTION

The Basidiomycota and the Ascomycota constitute the two largest fungal phyla and contain many of the most damaging crop pathogens (1). The dominant life phase for most basidiomycete species is dikaryotic, where two haploid nuclei coexist within one cell (2). To date, about 475 basidiomycete fungal genome sequences representing some 245 species are available in the public domain (as of September 2017 [https://www.ncbi.nlm.nih.gov/genome/]). These genome references are either representations of the haploid life stage of a species (3) or collapsed and mosaic assemblies of the dikaryotic state (4–7). Hence, information about the interhaplotype variation in dikaryotic Basidiomycota beyond single nucleotide polymorphisms (SNPs) and small insertions and deletions (indels) is very limited. The absence of haplotype-phased information limits the studies of genome architecture and evolution, particularly for the rust fungi of the order *Pucciniales*, many of which are extremely destructive pathogens of economically important crops, including cereals, coffee, and soybean (8–13).

Stripe, stem, and leaf rusts are the three rust diseases that impact wheat production, one of the most important staples in human diets. Of these, stripe rust caused by *Puccinia striiformis* f. sp. *tritici* is currently the most damaging disease, with estimated annual losses of $1 billion USD (14, 15). As a biotrophic pathogen, *P. striiformis* f. sp. *tritici* colonizes living hosts and extracts large amounts of nutrients from plant cells through specialized structures called haustoria. The large tax on host energy reserves caused by *P. striiformis* f. sp. *tritici* infection results in yield losses mostly associated with poor grain filling (16).

The full life cycle of *P. striiformis* f. sp. *tritici* involves asexual and sexual reproductive phases associated with the production of specific spore types (13, 16). The damage to wheat occurs during the asexual cycle and results from repeated infections throughout the growing season that cause exponential amplification of dikaryotic urediniospores. *P. striiformis* f. sp. *tritici* infects more than 30 varieties of *Berberis* spp. and *Mahonia* spp. to complete its full sexual life cycle, which involves four additional spore stages and sexual recombination during meiosis (17–19). Sexual reproduction is restricted geographically to the Himalayan region (Nepal, Pakistan, and China), where it leads to high levels of genetic diversity that are largely absent in other parts of the world. This makes the extended Himalayan region the center of *P. striiformis* f. sp. *tritici* diversity and the main source for new highly virulent *P. striiformis* f. sp. *tritici* isolates (12, 20).

Genetic resistance in the host plant, particularly race-specific resistance, is often used in the field to reduce damage by pathogenic rust fungi (21, 22). Race-specific resistance is generally conferred by dominant resistance (*R*) genes in the host, which recognize specific avirulence (*Avr*) alleles within the pathogen. Mechanistically, *Avr* alleles encode variants of virulence effector proteins, and the *R* gene typically encodes a nucleotide-binding leucine-rich repeat (NB-LRR) protein that detects the Avr protein within the infected plant cell. In the case of *P. striiformis* f. sp. *tritici*, more than 75 yellow rust resistance genes (*Yr*) have been cataloged to date. A given *P. striiformis* f. sp. *tritici* isolate has a characteristic spectrum of *Avr* alleles that can be distinguished on a set of wheat tester lines containing these *Yr* genes (23). The collective virulence phenotypes on such differential sets defines the *P. striiformis* f. sp. *tritici* pathotype. Wheat stripe rust epidemics are associated with the appearance of genetically novel pathotypes which are not recognized by currently employed *R* genes and hence grow on commercial wheat cultivars. As such, incursions of exotic stripe rust isolates with new virulence traits can play a role in disease outbreaks, for instance, the Warrior *P. striiformis* f. sp. *tritici* lineage, which invaded Europe in 2011, was highly successful because it was virulent on the wheat cultivars grown at that time (24, 25). In addition to this novel exotic incursion, it has been well-documented that *P. striiformis* f. sp. *tritici* rapidly evolved new virulence traits on a continental scale in Australia following its introduction in 1979 (26). However, the mechanisms underlying the evolution of these new pathotypes remain understudied, as no genetic locus contributing to the evolution of virulence has yet been identified in *P. striiformis* f. sp. *tritici*. While new combinations of alleles generated during sexual recombination can lead to the emergence of new pathotypes, the contributions of other genetic and molecular events to pathogen evolution during asexual reproduction are unclear. Presumably, the occurrence of mutations explains the loss of *Avr* specificities and the adaptation to otherwise-resistant wheat cultivars (13, 26).

Most agriculturally important fungi are haploid with small genomes (27). Rusts, on the other hand, are dikaryotic in the asexual phase and have expanded genomes with large amounts of repetitive sequence (6, 7). It is likely that the separation of rust genomes into two haploid copies contributes to their rapid evolution. Existing *P. striiformis* f. sp. *tritici* genome sequences suffer from the use of short-read sequencing technologies, which prevent characterization of individual haploid genomes, while the high percentage of repetitive DNA reduces the size of contigs that can be assembled (4, 5, 28). The overall similar gene content of each genome causes the reads from allelic variants to collapse upon assembly, producing a consensus sequence that loses haplotype (phasing) information. Read mapping to the consensus reference revealed that the two genomes are highly heterozygous for SNPs (5, 7), but differences in effector and gene content are undetectable. These problems can be addressed to some extent by using traditional Sanger long-sequence reads or strategies such as fosmid-to-fosmid sequencing (6, 7); however, these approaches are expensive. Opportunities to resolve the questions at higher resolution have arisen from new technologies that generate very long sequencing reads (>10 kb) (29, 30).

Here, we used long-read sequencing to provide a near-complete haplotype-phased genome assembly for an isolate representing the first pathotype of *P. striiformis* f. sp. *tritici* detected in Australia in 1979 (26). Our assembly provides the most complete *P. striiformis* f. sp. *tritici* genome reference to date, with over 97% of all basidiomycete benchmarking universal single-copy orthologs (BUSCOs) captured (31). In addition, phased haplotype information for over 92% of the genome enabled us to detect high interhaplotype diversity at the nucleotide and structural levels, which identified allelic variation and showed that 25% of all genes lack a clear allelic counterpart. We identified over 1,700 candidate effector genes which are more often spatially associated with each other and conserved BUSCOs than with repetitive elements. Nonallelic candidate effectors that lack counterparts in the alternate haploid genome region are less likely to be evolutionarily conserved in other rust fungi. Thus, the highly contiguous haplotype assembly has allowed discovery of novel genome features that may be linked to the rapid evolution of this devastating pathogen.

## RESULTS AND DISCUSSION

### Haplotype-aware genome assembly of an Australian *Puccinia striiformis* f. sp. *tritici* isolate.

The main aim of this study was to generate a high-quality reference genome for *P. striiformis* f. sp. *tritici*. For this purpose, we sequenced a single pustule isolate of the Australian founder pathotype *P. striiformis* f. sp. *tritici* 104E137A-, collected in 1982 (this strain is abbreviated *Pst*-104E). We sequenced 13 PacBio SMRT cells and obtained a total of 13.7 Gb of data with an average read length of 10,710 bases and a read length *N*_50_ of 15,196 bases (see Table S1A in the supplemental material). We assembled these data using the diploid-aware assembler FALCON-Unzip (29) to obtain a synthetic haplotype-phased reference genome. The FALCON-Unzip assembler is designed to phase structural variations and associated SNPs into distinct haplotype blocks. This process gives rise to a primary assembly (primary contigs) and linked haplotype blocks (haplotigs). The haplotigs represent the alternative genome structure with respect to primary contigs. FALCON-Unzip does not always link physically connected phase blocks, and primary contigs can represent sequences from either of the two haploid genomes (29).

10.1128/mBio.02275-17.7 TABLE S1  (A) Summary table of PacBio genome sequencing using 13 SMRT cells. (B) Annotation of gene classes on primary contigs and haplotigs. For each category, the number of proteins and the percentages of proteins with a hit in each category are given. The first number in each column indicates the total number of proteins, and the second number is the percentage within each category. (C) Summary of interhaplotype allele analysis. The table summarizes the allele and conservation state of *Pst*-104E genes. Primary contigs and haplotigs were treated as two representative units for orthology analysis in Proteinortho with a synteny flag. The three major categories are highlighted in bold. In the cases of alleles, they can be subdivided into three categories, as illustrated in Fig. S4. Superscript letters A, B, and C correspond to Fig. S4, panels A, B, and C. The numbers in brackets for haplotype singletons is the number of singletons given by Proteinortho without filtering for unphased gene models based on genome coverage analysis. True-haplotype singletons are located in phased regions of the genome. (D) Number of total proteins and predicted secreted proteins in publicly available cereal rust genomes. Numbers in brackets indicate the percentages of proteins that are predicted to be secreted in each proteome using SignalP3. (E) Summary table of genes located in indicated expression clusters with regard to EffectorP, nuclear localization, and apoplastic localization prediction. We used EffectorP (63), Localizer (64), and ApoplastP (122) for predictions. (F) Summary of the allele state and orthologous expression patterns of secreted protein-coding genes located on primary contigs. The top half of the table shows how many genes are within each cluster and, of those, how many are allelic, interhaplotype paralogs, or singletons. The bottom half of the table shows how the expression of alleles of genes located on primary contigs cluster in the haplotig gene expression analysis. (G) Summary of the allele state and orthologous expression patterns of secreted protein-coding genes located on haplotigs. The top half of the table shows how many genes are within each cluster and, of those, how many are allelic, interhaplotype paralogs, or singletons. The bottom half of the table shows how the expression of alleles of haplotig genes cluster in the primary gene expression analysis. (H) Changes in genome size and contig number at different steps in the assembly process. Download TABLE S1, DOCX file, 0.03 MB. Copyright © 2018 Schwessinger et al. 2018 Schwessinger et al. This content is distributed under the terms of the Creative Commons Attribution 4.0 International license.

Previous unphased *P. striiformis* f. sp. *tritici* genome assemblies ranged in size from 53 to 115 Mb (4, 5, 7, 28). In an attempt to reconcile the differences in reported genome sizes, we used GenomeScope to estimate the haploid genome size, using *k*-mer frequencies (30-mers) in two Illumina short-read data sets of *Pst*-104E (32). Based on this analysis, we estimated a haploid genome size of 68 to 71 Mb, with a heterozygosity (SNPs and indels) rate of approximately 1.2%. We assembled our long-read data into 156 primary contigs with a total length of 83 Mb after manual curation. The corresponding phased haplotype blocks were contained in 475 haplotigs with a total size of 73 Mb (Table 1).

**TABLE 1:** Summary of *Pst*-104E genome assembly and annotation^a^

Parameter	Primary assembly		Haplotype assembly
	Primary contigs with haplotigs	Primary contigs without haplotigs	Haplotigs
No. of contigs	99	57	475
No. of bases	79,770,604	3,585,012	73,478,481
TE coverage (%)	53.72	67.17	52.82
No. of genes	15,303	625	14,321
Avg gene length	1,210	1,290	1,189
Avg no. of introns/gene	3.45	2.70	3.42
No. of genes/10 kb	1.92	1.74	1.95
No. of BUSCOs	1395	49	1,293
No. of BUSCOs/10 kb	0.17	0.14	0.18
No. of candidate effectors^b^	1,523	49	1,390
No. of candidate effectors/10 kb	0.19	0.14	0.19

a Summary statistics for the genome assembly according to the three different contig categories as described in the main text.

b Candidate effectors were predicted based on the machine-learning algorithm EffectorP and transcriptional upregulation during infection of wheat, as described in the text.

**FIG 1:**
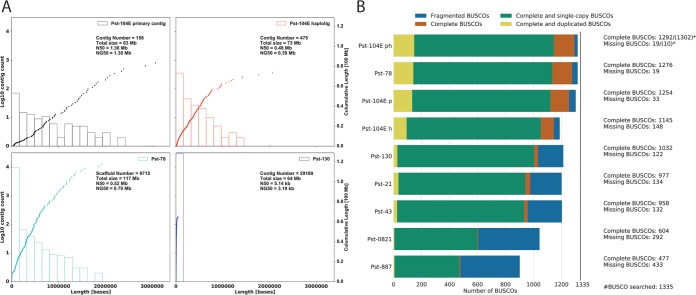
The *Pst*-104E genome assembly is highly contiguous and complete. (A) Comparison of the *Pst*-104E primary and haplotig assemblies with the two most complete publicly available *P. striiformis* f. sp. *tritici* genome assemblies, *Pst*-78 and *Pst*-130. The histograms and the left *y* axis show log_10_ counts of contigs within each size bin. The dots and the right *y* axis show the cumulative sizes of small to large sorted contig lengths. Each dot represents a single contig of the given size, shown on the *x* axis. Each plot also shows the number of contigs or scaffolds, total assembly size, *N*_50_ of the assembly, and NG_50_ assuming a genome size of 85 Mb. NG_50_ is the *N*_50_ of an assembly considering the estimated genome size instead of the actual assembly size. This enables comparisons between different-sized assemblies. (B) Genome completeness was assessed using benchmarking universal single-copy orthologs (BUSCOs) for *Basidiomycota* (odb9) as proxy. The graph shows BUSCO results for *Pst*-104E primary (p), haplotig (h), and nonredundantly combined (ph) assemblies, in comparison to all publicly available *P. striiformis* f. sp. *tritici* genome assemblies with gene models, including *Pst*-78, *Pst*-130, *Pst*-21, *Pst*-43, *Pst*-0821, and *Pst*-887. The analysis was performed on the protein level, using publicly available gene models. An asterisk indicates the actual number of identified BUSCOs for the complete *Pst*-104E ph assembly before filtering gene models for similarity with genes related to transposable elements.

These assembly statistics are a vast improvement over information available from previous assemblies in terms of connectivity and number of contigs (Fig. 1A). The primary assembly has a contig *N*_50_ of 1.3 Mb, compared to a scaffold *N*_50_ of 0.5 Mb for *Pst*-78 or contig *N*_50_ of 5.1 kb for *Pst*-130, often referred to as the reference genome (4, 25, 28). In addition, we identified 1,302 (97.5%) of the 1,335 benchmarking genes (BUSCO v2; http://busco.ezlab.org/v2) (31) that are highly conserved in basidiomycetes, with only 10 (0.7%) missing in our combined assembly before filtering for genes related to transposable elements (TE). Our final assembly had 1,292 (96.8%) complete BUSCOs, with 19 (1.4%) missing. Compared to the wide variation in BUSCOs that identified from previous assemblies, ranging from 35.7% for *Pst*-887 to 95.6% for *Pst*-78 (Fig. 1B). In summary, our assembly currently represents the most complete *P. striiformis* f. sp. *tritici* reference in terms of contiguity, haplotype-phased information, and gene content. This advance provides a new resource to investigate genome architecture and interhaplotype variation for this dikaryotic plant pathogen.

### High levels of interhaplotype block variation.

The *Pst*-104E primary assembly covers 83 Mb in a total of 156 primary contigs. Within this assembly, 99 primary contigs (~80 Mb) are associated with 475 haplotigs (~73 Mb), representing phased information for 92% of the primary contigs. These primary contigs are referred to as primary contigs with haplotigs. Overall, short-read mapping coverage analysis strongly supported our genome assembly. When we mapped short reads against the primary assembly, we observed a bimodal distribution of coverage, with a haploid genome coverage around ~60-fold and a diploid genome coverage of ~120-fold (Fig. S1A). Regions with ~60-fold coverage are sequences that are distinct enough between the two haplotypes that only short reads originating from these specific sequences can be mapped. Regions with ~120-fold coverage are sequences that are similar enough in the two haplotypes that short reads from both haplotypes collapse on the primary contig sequence when mapped against primary contigs only.

10.1128/mBio.02275-17.1 FIG S1  Short-read genome coverage analysis suggests high levels of haplotype-specific sequences. (A to C) Illumina short-read coverage plots for the color-coded genome regions mapped against primary contigs only. (D to G) Illumina short-read coverage plots for the color-coded genome regions mapped against combined primary contigs and haplotigs. The peak at ~120-fold coverage represents diploid genome regions, which are similar for both haplotypes. The peak at ~60-fold coverage represents haploid genome regions, which are specific to one haplotype. Most regions on primary contigs that do not align with a corresponding haplotig display haploid genome coverage (C and G), indicating haplotype-specific sequences. Plots are trimmed to the 99th percentile to remove high-coverage outliers, such as collapsed repeat regions, for visualization purposes. Download FIG S1, TIF file, 6.8 MB. Copyright © 2018 Schwessinger et al. 2018 Schwessinger et al. This content is distributed under the terms of the Creative Commons Attribution 4.0 International license.

In contrast, when reads were mapped against both primary contigs and haplotigs, we found haplotigs and phased primary contig regions, which align haplotigs, displayed ~60-fold coverage (Fig. S1E and F). These are regions of the *Pst*-104E genome that are phased into two haplotype blocks. In addition, primary contig regions that lack an associated haplotig display mostly ~60-fold coverage (Fig. S1C and G), suggesting that these are largely sequences specific to one haplotype and not collapsed highly similar regions of corresponding chromosome copies. Only a minor fraction of primary contigs show ~120-fold coverage (Fig. S1D and G) when mapped against primary contigs and haplotigs, indicating the presence of a low residual of unphased sequences in our assembly.

**FIG 2:**
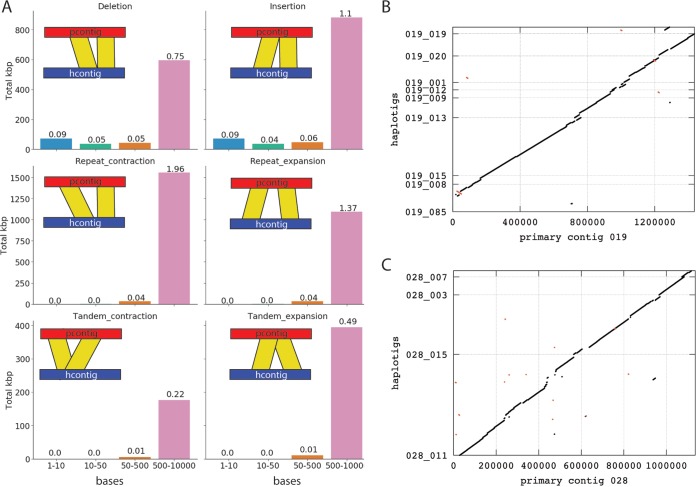
The *Pst*-104E genome is characterized by high levels of interhaplotype variation. (A) Summary of interhaplotype variation between primary contigs and their respective haplotigs, analyzed using Assemblytics. Each plot indicates the number of bases that are spanned by the specific variation category, which is illustrated by a cartoon. The number labeling each histogram represents the percentage of the total size of primary contigs with haplotigs that are contained within this variation type and size bin. (B and C) Two representative whole-genome alignments of primary contigs 019 and 028 with their respective haplotigs. This illustrates the large-scale variations summarized in panel A.

Of the 57 primary contigs (~3.6 Mb) without associated haplotigs (Table 1), 51 (~3.4 Mb) are likely single-haplotype-specific sequences, because they display similar mean read coverage (~60-fold) to phased haploid regions of the genome (Fig. S1). This high level of phasing enabled us to investigate interhaplotype variation on a whole-genome scale. Previous studies using Illumina short reads mapped against the consensus merged haplotype assemblies estimated *P. striiformis* f. sp. *tritici* interhaplotype variation based on heterozygous SNPs between 0.5% and 1% (5, 7, 28). Taking a similar approach, we identified approximately 0.5% (416,460 heterozygous SNPs) of the genome as variable when mapping lllumina short reads against primary contigs only. However, we estimated a dramatically higher level of interhaplotype variation when using this phased assembly. For this analysis, we aligned all haplotigs with their corresponding primary contigs and estimated variations by using Assemblytics (33, 34). Assemblytics defines six major categories of structural variations, including insertions and deletions, tandem repeats identified by overlapping alignments and other types of repeats suggested by gapped nonunique contig alignments (see Fig. 2A for illustration of the six different variant categories) and divides these according to size into bins (Fig. 2A). This analysis revealed that structural variation comprised 6.4% (~5.10/79.77 Mb) of the primary assembly space compared to corresponding haplotigs (Fig. 2A) (33). The variation between two primary contigs and their respective haplotigs is illustrated in the dot plots shown in Fig. 2B and C, with large-scale inversions, deletions, and insertions in haplotigs associated with two primary contigs. It is likely that the actual difference between the two haplotypes is higher than the estimated 6.4%, because calculations were restricted to a maximal variant size of 10 kb and did not include primary contigs without haplotigs, which account for another ~3.6%. Overall, the dramatic difference in estimated interhaplotype variation between previous assemblies (5, 7, 28) and short-read-based prediction programs (32) is likely caused by the fact that most of the observed variations are contained in size bins greater than 500 bases, which are not detectable with Illumina short-read data and highly fragmented assemblies.

### **Over half of the** ***Pst***-**104E genome is covered by repetitive sequences.**

We annotated primary contigs and haplotigs independently based on our observations of high levels of heterozygosity between the two (Fig. 2; Fig. S2). We first identified and classified TEs by using the REPET pipeline (35) to the order level, based on the Wicker classification (36). We further transferred superfamily annotations from the underlying BLAST (37) hits if they agreed with the REPET annotations and with each other. There was no major difference between TE coverage of primary contigs (54%, ~45 Mb) and haplotigs (53%, ~39 Mb) (Fig. S2). However, primary contigs that lacked haplotigs had a larger proportion of TEs, with a total coverage of 67%, which may explain their increased fragmentation, reduced contig length, and inability to assign haplotigs (Table 1). The composition of TE superfamilies on primary contigs versus haplotigs was very similar (Fig. S2). Both retrotransposons (class I) and DNA transposons (class II) cover 30% of the genome each (note that distinct TEs belonging to different categories can overlap). For class I transposons, the long terminal repeat (LTR) order was the most prominent, with ~27% coverage, and within this order elements from the Gypsy and Copia superfamilies were most prominent. The only other class I orders with greater than 1% genome coverage were LARD and DIRS elements. Class II elements were dominated by TIR elements, with a genome coverage of ~20%, with significant contributions of elements belonging to the hAT, MuDR, PIF-Harbinger, Tc1-Mariner, and CATCA superfamilies. More than 6% of the genome was covered by class II elements that could not be classified below the class level and showed no homology to previously identified TEs. This was in contrast to the minimal coverage by unclassifiable class I elements (0.05%).

10.1128/mBio.02275-17.2 FIG S2  Over half of the *Pst*-104E genome is covered by repetitive elements. (A and B) Repetitive element annotation on primary contigs (83 Mb) (A) and on haplotigs (73 Mb) (B). Top panels show percentages of genome coverage for all repetitive elements and different subcategories. These include TEs of class I (RNA retrotransposons) and class II (DNA transposons), simple sequence repeats (SSR), and unclassifiable repeats (no Cat). Middle and bottom panels show percentages of genome coverage of class I and class II TEs categorized to class, order, and superfamily levels wherever possible. Repetitive elements were identified using the REPET pipeline, and classifications were inferred from the closest BLAST hit (see Materials and Methods in the text). Download FIG S2, TIF file, 1.5 MB. Copyright © 2018 Schwessinger et al. 2018 Schwessinger et al. This content is distributed under the terms of the Creative Commons Attribution 4.0 International license.

Overall, this is the highest number of identified transposable elements detected in any *P. striiformis* f. sp. *tritici* genome assemblies so far, as previous reports varied from 17% to 50% (4, 7, 28). Such an increased content of identified transposable elements is likely due to the increased contiguity and the absence of any unidentified bases (Ns) in our assembly (Fig. 1).

Next, we reasoned that younger, less divergent TEs are mostly likely to contribute to current genome evolution. Therefore, we estimated TE ages on primary contigs, which are more contiguous than haplotigs, based on their divergence from the consensus sequence of each element (Fig. S3A and B; see also File 1 in the information available on our study’s github page at https://github.com/BenjaminSchwessinger/Pst_104_E137_A-_genome) (38). This enabled us to investigate how much of the genome is covered by relatively young TEs (<100 Mya in our approximation) with high copy numbers (>50 copies) (Fig. S3C). The genome coverage of these younger high-copy-number TEs followed the overall coverage analysis closely (Fig. S2B and C and S3C). Class I LTR elements, especially Copia and Gypsy superfamily members, and class II elements belonging to the TIR order and unclassified class II elements likely contribute to current genome evolution. In the future, the availability of further high-quality genome assemblies for rust fungi will provide greater insights into TE evolution in *Pucciniales* and their contribution to genome evolution.

10.1128/mBio.02275-17.3 FIG S3  Approximate age of TEs found on primary contigs. (A and B) Estimated ages of class I (A) and class II (B) elements at the superfamily level for TEs with more than 5 copies. (C) Genome coverage per TE superfamily of elements with an approximate age of less than 100 Mya and more than 50 copies. These TEs are most likely to contribute to current genome evolution. Bars are color-coded according to the order level. Download FIG S3, TIF file, 1 MB. Copyright © 2018 Schwessinger et al. 2018 Schwessinger et al. This content is distributed under the terms of the Creative Commons Attribution 4.0 International license.

### High levels of interhaplotype structural variation lead to variable gene content between primary contigs and haplotigs.

We also annotated gene models on primary contigs and haplotigs independently by using extensive sets of newly generated and publicly available transcriptome sequencing (RNA-seq) data (39). This is in contrast to previously published *P. striiformis* f. sp. *tritici* genomes that were annotated nearly exclusively using *ab initio* gene-finding approaches without gene expression data (4, 5, 7, 28). The newly generated RNA-seq data sets were obtained from dormant and germinated urediniospores, wheat leaf tissue 6 and 9 days postinfection (dpi), and haustoria-enriched fractions. These data sets were complemented by publicly available RNA-seq data from germinated spores and infected wheat tissue sampled at 13 different time point-plant genotype combinations (39). We used these extensive expression data in a comprehensive genome annotation pipeline (40–44) and identified 15,928 and 14,321 gene models on primary contigs and haplotigs, respectively, after filtering for genes related to TE function (Table 1; see also Table S1B) (45, 46). The protein sequences of these genes were functionally annotated using a number of bioinformatic tools (Table S1B; see also File 2 at our github website, as described above for File 1 and reported in our “Data Availability” section at the end of the Materials and Methods section) (31, 47–51). We obtained very similar annotation levels for primary contigs and haplotigs with about 52% of all proteins having at least one functional annotation in the following categories; GO terms, InterPro match, Pfam domain, EggNog term, KEGG pathway annotation, Merops catalytic domain, or carbohydrate hydrolyzing enzymatic domains (CAZy) (31, 47–51). The level of functional annotation for *P. striiformis* f. sp. *tritici* proteins identified as BUSCO orthologs was near complete with only three proteins in total (<0.1%) lacking any functionally recognizable domain (Table S1B). This pattern was reversed when characterizing candidate effectors (see identification below) as approximately 83% of all proteins lacked a conserved functional domain.

**FIG 3:**
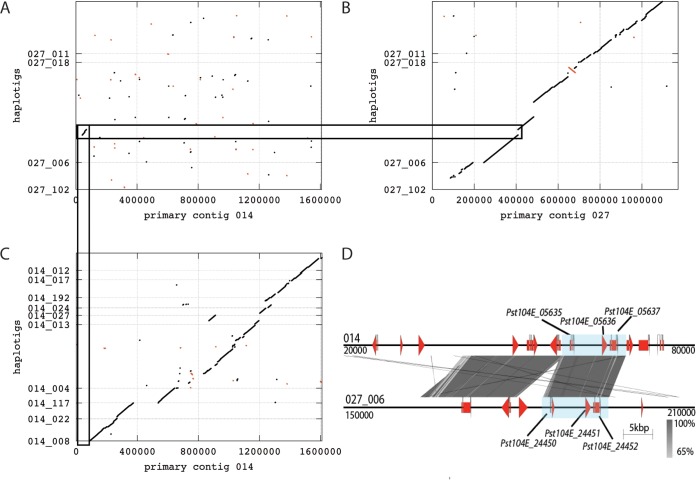
Allele transposition in the *Pst*-104E genome. **(**A to C) Dot plots of whole-genome alignments generated using the mummer toolset, where the *x* axis represents primary contig and the *y* axis shows the haplotig sequence. (A) The whole-genome alignments of haplotigs_027_xxx to primary contig 014. (B) The whole-genome alignment of haplotigs_027_xxx to primary contig 027. (C) The whole-genome alignment of haplotigs_014_xxx to primary contig 014. Black lines indicate alignments in the forward direction, and red lines indicate alignments in the reverse direction in the haplotig sequence. The black rectangles highlight an ~40-kb region in haplotig_027_006 that does not align to primary contig 027 yet aligns to a region in primary contig 014, which is not covered by an associated haplotig of 014. (D) Microsynteny analysis of this extended region, with primary contig 014 on top and haplotig_027_006 on the bottom. Gene models identified as alleles are labeled with their locus tag and shaded with a light blue background. Vertical gray shading illustrates the blastn identity between sequences on both contigs, according to the scale shown in the right bottom corner next to the sequence scale bar. Start and stop positions for each contig sequence are given at the start and the end of each contig.

Overall, the haplotype-phased assembly did not show biased distribution of any particular gene annotation group (Table S1B); this is consistent with the high level of haplotype phasing. This encouraged us to investigate the relationship between the two haplotype-phased block assemblies (primary contigs compared to haplotigs) in terms of gene content. One must keep in mind that these two assemblies do not actually represent the true haploid genomes, because of potential haplotype switching between primary contigs and haplotigs and the inability to assign independent contigs to a specific haploid genome copy (29). However, a relational comparison between the two assemblies is still valuable in order to investigate the approximate interhaplotype gene diversity. Therefore, to simplify the analysis, we treated primary contigs and haplotigs as two representative genetic units. We used Proteinortho in synteny mode to identify allele pairs between the primary contigs and haplotigs (52). We identified a total of 10,921 potential syntenic allele pairings, including 10,785 primary proteins and 10,860 haplotig proteins (Table S1C; see Files 3 and 4 at our study’s github repository for allelic variation comparisons). Of these, 9,756 were properly paired where the haplotig gene models were located on an associated haplotig that overlapped with the primary gene model when targeted whole-genome alignments were performed (Fig. S4A and Table S1B). These correspond to “classic” alleles in a diploid organism. Another 450 pairs were not directly linked, as the haplotig containing the allelic ortholog did not overlap with the primary gene model, although it was associated with the primary contig (Fig. S4B; File 3). These may be simple rearrangements linked to inversions or repeat duplications. A further 715 pairs were completely unlinked, as the allele-containing haplotig was not associated with the respective primary contig in our assembly (Fig. S4C; File 4). We randomly selected 176 of these loci and investigated them manually by whole-genome alignment of haplotigs to primary contigs, followed by microsynteny analysis of the identified gene loci (34, 53, 54). An example of this analysis is illustrated in Fig. 3. In this case, an ~40-kb region present in both primary contig 014 and haplotig 027_006 showed microsynteny for three genes each, namely, *Pst104E_05635-05637* and *Pst_104E_24450-24452*, respectively (Fig. 3D), while the overall macrosynteny was not conserved (Fig. 3A to C). This may have been caused by genetic transposition of the identified region from the chromosomal region corresponding to a haplotig that fully aligned with primary contig 014 into the sequence of the chromosomal region corresponding to haplotig 027_006. We found support for such allele transposition, either via cut-and-paste or copy-and-paste mechanisms, in 71/176 cases. The remaining cases could not be categorized confidently and may represent complex genomic regions, genetically linked contigs that were broken up during the assembly process, gene duplication events, or misassemblies. Based on this manual inspection, we estimated that approximately 280 loci ([71/176] × 715 total pairs) contain alleles that might be rearranged in one of the two haploid genomes. We identified a further 912 loci that clustered at the protein level, yet their genomic location was not syntenic between the two haplotype-phased block assemblies (see File 5 at our study’s github repository). We refer to these genes as interhaplotype paralogs. In summary, our findings suggest that over 3% (~1,192/30,249) of all genes are closely related at the protein level but do not reside in regions displaying macrosynteny.

**FIG 4:**
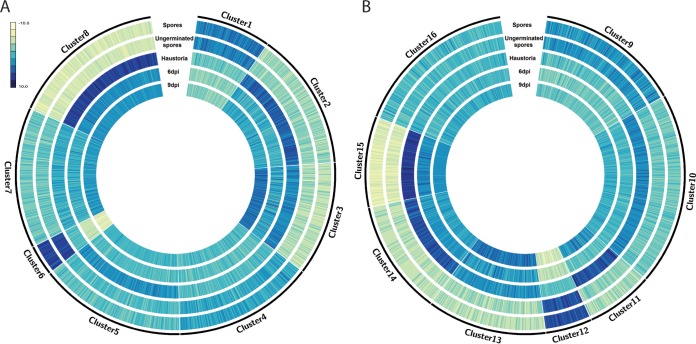
Identification of candidate effectors based on detailed expression analysis of secreted proteins of both *Pst*-104E assemblies. (A) Clustering of *Pst*-104E secretome expression profiles for genes located on primary contigs. Blue color intensity indicates the relative expression level based on rlog-transformed read counts in spores, germinated spores, haustoria, and in wheat tissue at 6 and 9 days postinfection. For example, cluster 8 shows the lowest relative expression in spores and the highest in haustoria, compared to the other clusters. (B) Clustering of *Pst*-104E secretome expression profiles for genes located on haplotigs.

10.1128/mBio.02275-17.4 FIG S4  Illustration of the three observed allele configuration categories in the *Pst*-104E haplotype-phased assembly. (A) “Classic” alleles identified by Proteinortho for which the gene models of allele pairs are located in regions of primary contigs and associated haplotigs that overlap in whole-genome alignments. (B) Alleles detected on nonoverlapping associated haplotigs. These are alleles identified by Proteinortho for which the gene models of allele pairs are located in regions of primary contigs and associated haplotigs that do not overlap in whole-genome alignments. (C) Alleles detected on nonassociated haplotigs and primary contigs that might represent long-range transpositions. These are alleles identified by Proteinortho for which the gene models of allele pairs are located on haplotigs that are not associated with the primary contig. See also Fig. 4 for a detailed illustration of one example. Download FIG S4, TIF file, 1.4 MB. Copyright © 2018 Schwessinger et al. 2018 Schwessinger et al. This content is distributed under the terms of the Creative Commons Attribution 4.0 International license.

We identified 4,761 primary and 2,931 haplotig genes that did not cluster at the protein level when we used Proteinortho, and hence these may represent singletons, with singletons defined as genes of a diploid/dihaploid organism that lack alleles or interhaplotype paralogs (Table S1C). Of the 4,761 primary genes, 663 were located in regions where the assembly was not haplotype phased based on coverage analysis using Illumina short-read data (File 6). From these results, we identified 7,029 true singletons (File 7) when we compared both haplotype-phase block assemblies, and 1,506 of these singletons are referred to as single haplotype genes (File 8) because they lacked any BLAST hit (blastn, e value of <0.01) when we used the gene sequence as a query against the alternate haplotype-phase block sequence. These single-haplotype genes are often linked in clusters, because for 1,164 single-haplotype genes, at least 1 of their nearest neighbors is also a haplotype-specific gene, compared to 212 of an equally sized random subsample of all genes (Fisher’s exact test, *P* ≈ 2.3 × 10^−109^). Similarly, 1,492 haplotype-specific genes are located in regions where primary contigs and associated haplotigs do not align, indicating haplotype-specific regions. Single-haplotype genes are highly enriched in these regions, as only 251 of an equally sized random subsample of all genes displayed a similar location (Fisher’s exact test, *P* ≈ 4.5 × 10^−265^). Taken together, these findings suggest that there are numerous large presence/absence structural polymorphisms between the two haploid genomes that can span multiple adjacent genes and therefore contain many of the haplotype-specific genes. To study the overall conservation of these single-haplotype genes, we queried them against the EnsemblFungi cDNA and NCBI nr databases (blastn, e value of <0.01) (55, 56). Out of 1,506 genes, 1,424 had at least one significant hit in either database, with the top hits in all cases being fungal sequences. The remaining 82 genes lacked any sequence homology to known fungal genes. These genes were significantly shorter compared to all genes (mean lengths of 538 bases versus 1,538; two-sided Student’s *t* test, *P*  ≈ 2.38e^−07^). We identified expression evidence for 27/82 of these genes, including 7 of 10 predicted candidate effectors. This is consistent with observations in other fungi for which isolate-specific genes tend to be shorter and are expressed at lower levels than genes that are conserved between isolates (57). Overall, the high levels of nonallelic genes (~25%) and single-haplotype genes (~5%) illustrate that the large interhaplotype polymorphism on the nucleotide and structural levels (Fig. 2 and 3B and C) results in significant differences in gene content.

### Candidate effector gene prediction using machine learning and *in planta* expression data.

The diversity of plant pathogen effectors makes them impossible to identify based on protein sequences alone (58). Only a small number of effectors have thus far been confirmed in rust fungi, namely, AvrP123, AvrP4, AvrL567, AvrM, RTP1, PGTAUSPE-10-1 (59), AvrL2 and AvrM14 (60), PstSCR1 (61) and PEC6 (62). At the sequence level, effectors do not share common domains or motifs, apart from the presence of a signal peptide. To predict candidate effectors in *Pst*-104E, we utilized a combination of gene expression analysis and machine learning methods. First, we predicted fungal rust secretomes based on a protocol optimized for recovering fungal candidate effectors (63). We observed large differences in secretome sizes across rust proteomes, e.g., the stripe rust isolate *Pst*-887 had a small secretome compared to *Pst*-104E (Table S1D). Overall the number of secreted proteins appeared to correlate with completeness of *P. striiformis* f. sp. *tritici* genome assemblies based on BUSCO analysis (Fig. 1B; Table S1D). This implies that it is difficult to perform comprehensive orthology analyses between current *P. striiformis* f. sp. *tritici* assemblies, given that many appear to be incomplete in terms of BUSCOs and therefore are likely incomplete for other gene families also, including secreted proteins.

To predict candidate effectors, we used the machine-learning approach EffectorP on all secreted proteins without predicted transmembrane domains (63). Overall, we identified 1,069 and 969 candidate effectors from primary contigs and haplotigs, respectively (File 9). We complemented this *in silico* approach with a detailed expression analysis of *Pst*-104E genes that encode secreted proteins. We used gene expression data and *k*-means clustering to predict clusters in the secretome that are differentially expressed during infection and exhibit similar expression profiles (Fig. 4; File 10). For the primary contigs of *Pst*-104E, this resulted in eight predicted clusters. The expression profiles of three clusters (clusters 2, 3, and 8) resembled the expected expression patterns of haustorially delivered cytoplasmic rust effectors, namely, high expression in haustorial tissue and at the infection time points of 6 and 9 dpi, as well as low expression in spores (Fig. 4A). In total, there are 809 genes in clusters 2, 3, and 8, of which 306 (~38%) were also identified by EffectorP as candidate effectors (Table S1E). Upon closer inspection of primary contig expression patterns, cluster 8 in particular exhibits the highest overall haustorial expression and overall lowest expression in spores, indicating it is likely to contain cytoplasmic effectors. Interestingly, while cluster 8 shows the lowest percentage of EffectorP-predicted candidate effectors (26%), it has the highest percentage of proteins with a predicted nuclear localization signal (NLS) (Table S1E) (64). We also observed that proteins in cluster 8 are mostly larger (average length of 410 amino acids [aa]) than other known rust effectors (the largest is AvrM, at 314 aa), which might indicate that *P. striiformis* f. sp. *tritici* utilizes a class of larger effector proteins that target host nuclei. Similarly, oomycete pathogens secrete a class of cytoplasmic effectors called Crinklers that carry NLSs (65, 66), but these are not candidate effectors predicted by EffectorP, possibly due to their larger size. Therefore, we included both *in planta*-upregulated secreted proteins as well as EffectorP-predicted proteins as candidate effectors. In total, we identified 1,572 candidate effectors on primary contigs when we combined predictions based on *in planta* expression analysis and EffectorP. We identified similar expression patterns for secreted proteins on haplotigs. Clusters 11, 13, 14, and 15 shared a similar expression profile to clusters 2, 3, and 8 and contained 673 genes (Table S1F and G). Of these, 234 (~37%) were also identified by EffectorP, amounting to a total of 1,388 candidate effectors on haplotigs. Overall, we identified a set of 1,725 nonredundant candidate effectors, counting allelic candidate effector pairs only once, when we combined all candidate effectors on primary contigs and haplotigs (File 11).

### Candidate effector genes are spatially associated with conserved genes and with each other.

**FIG 5:**
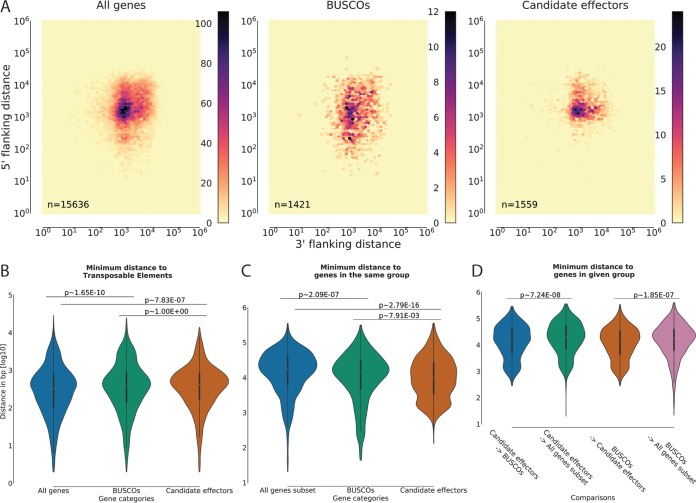
Candidate effector genes are spatially associated with conserved genes and with each other. (A) Nearest-neighbor gene distance density hexplots for three gene categories, including all genes, BUSCOs, and candidate effectors. Each subplot represents a distance density hexplot with the log_10_ 3′-flanking and 5′-flanking distance to the nearest-neighboring gene plotted along the *x* axis and *y* axis, respectively. (B) Violin plots for the log_10_ distance to the most proximal transposable element for genes in each category without allowing for overlap. (C) Violin plots for the log_10_ distance to the most proximal gene in the same category for subsamples of each category equivalent to the smallest category size (*n* = 1,444). (D) Violin plots for the minimum distance (log_10_) of candidate effectors and BUSCOs to each other or a random subset of genes (*n* = 1,444). The *P* values for panels B, C, and D were calculated using the Wilcoxon rank-sum test after correction for multiple testing (Bonferroni; alpha = 0.05) on the linear distance in bases.

**FIG 6:**
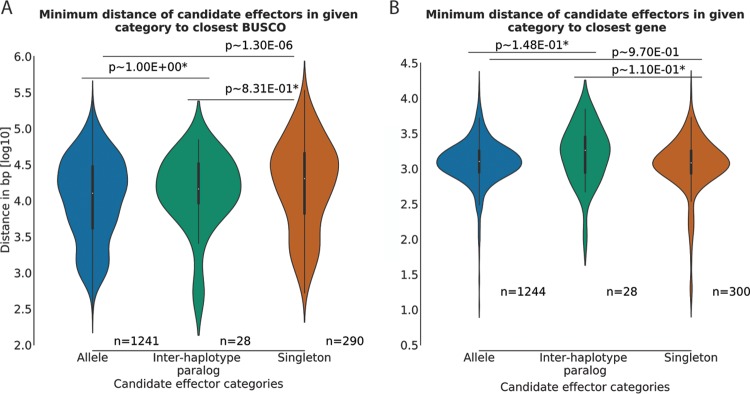
The candidate effector allele status influences association with conserved genes. (A) Violin plots for the log_10_ distance to the most proximal BUSCO for candidate effectors in each category. The Kruskal-Wallis one-way analysis of variance of all three categories showed a significant difference between the three samples (*P*, ~2.36e^−06^). (B) Violin plots for the log_10_ distance to the most proximal gene for candidate effectors in each category. The Kruskal-Wallis one-way analysis of variance of all three categories showed no significant difference between the three samples (*P*, ~0.08). The *P* values in panels A and B were calculated using the Wilcoxon rank-sum test after correction for multiple testing (Bonferroni; alpha = 0.05) on the linear distance in bases. *, Wilcoxon rank-sum test comparisons with interhaploid genome paralogs lacked statistical power due to the small sample size (*n* = 28).

For many filamentous plant pathogens a “two-speed genome” has been suggested to contribute to rapid evolution in terms of candidate effector variability (67). For example, in fungal plant pathogens such as *Fusarium oxysporum* and *Verticillium dahliae*, lineage-specific genomic regions and/or dispensable chromosomes are enriched for TEs and candidate effector genes (68, 69, 70). In several *Phytophthora* spp., candidate effectors have been reported to localize in gene-sparse, TE-rich regions, which show signs of accelerated evolution (67, 71). It is not known if rust genomes have a comparable genome architecture that facilitates rapid evolution of candidate effector genes. Therefore, we investigated the genomic location of candidate effectors in relation to several genomic features, including TEs, neighboring genes, BUSCOs, other candidate effectors, and AT content (Fig. 5 and 6; Fig. S5 and S6). We focused mostly on candidate effectors on primary contigs, because the primary assembly is far more contiguous than its haplotigs, thereby facilitating our analysis (Fig. 1). In addition, we made use of our haplotype-phased assembly and investigated if allelic candidate effector variants show features distinct from haplotype singletons. In all cases, we used a random subset of genes and BUSCO gene sets as control groups. We envisioned BUSCO genes as a particularly well-suited control group, as these are conserved within the phylum of *Basidiomycetes* (31) and can therefore be considered part of the *P. striiformis* f. sp. *tritici* core genome. On the contrary, candidate effector genes are reported to be more specific on the class, species, or isolate level (6, 73). This observation also holds true for *Pst*-104E, because we only observed 40 BLAST hits outside the class of *Pucciniomycetes* for 1,725 nonredundant candidate effectors when we used EnsemblFungi cDNA as the reference (blastn, 1e−5).

We first tested if candidate effectors are located in gene-sparse regions compared to all genes or BUSCOs. For this analysis, we generated density plots using the distances from the 5′ and 3′ ends of each gene to its closest neighbor in either direction (67). When we compared gene distance density hexplots, we observed very similar distributions between candidate effectors and all genes. Candidate effectors in general did not appear to be located in gene-sparse regions, and neither did BUSCOs (Fig. 5A). Similar effects have been reported for other rust species, such as the oat crown rust pathogen *Puccinia coronata* f. sp. *avenae* (74). Next, we tested if candidate effectors are linked to TEs, as observed for other plant-pathogenic fungi (75). We compared the minimum distance of all genes, BUSCOs, and candidate effectors to TEs. Candidate effectors globally did not display a preferential association with TEs compared with genes in general (Fig. 5B). However, on close examination of the relative spatial distribution of TEs, candidate effectors, and BUSCOs on the 30 largest contigs, we could identify some regions where candidate effectors are closely associated with TEs (Fig. S5). The observation that candidate effectors are not associated globally with TEs is consistent with reports of other rust fungi, including *P. coronata* f. sp. *avenae*, *Puccinia graminis tritici*, and *Melampsora larici-populina* (6,74). In the case of *P. striiformis* f. sp. *tritici*, we aim to address the question of the involvement of TEs in the evolution of novel virulences by resequencing *Pst*-104E mutant progeny with distinct virulence profiles collected in Australia between 1980 and 2003 (26).

10.1128/mBio.02275-17.5 FIG S5  Spatial correlation of AT richness, repeat coverage, candidate effectors, and BUSCOs. The circa plot shows the 30 largest primary contigs, ordered according to size. The total length shown is greater than 50 Mb. See the key in the figure itself for details of each track. Lines with reverse arrowheads illustrate regions in the *Pst*-104E genome where candidate effectors cluster in repeat-poor regions. Lines with round arrowheads illustrate regions in the *Pst*-104E genome where candidate effectors appear to cluster with each other close to repeat-dense regions. RE, repetitive element. Download FIG S5, TIF file, 7.8 MB. Copyright © 2018 Schwessinger et al. 2018 Schwessinger et al. This content is distributed under the terms of the Creative Commons Attribution 4.0 International license.

10.1128/mBio.02275-17.6 FIG S6  Candidate effector genes are clustered. (A) Illustration of the clustering approach. In this example, genes 1 to 3 in the black category are clustered together, as each is separated by less than 12 kb from their closest neighbor of the same category. Gene 4 is not part of the cluster, as it is more than 12 kb away from gene 3. The clustering algorithm is oblivious to the fact that gene 1 from the red category is interspersed between genes 2 and 3 of the black category. This cluster thus contains 3 black genes. (B) Clustering of equal numbers of genes (*n* = 1,444) using a maximum distance cutoff of 12 kb. The *y* axis represents different gene categories, and the *x* axis represents the number of genes linked by a minimum distance of 12 kb. The numbers next to the circles indicate the total number of genes in each cluster size bin. The circles are drawn in proportion reflecting this number. Download FIG S6, TIF file, 0.8 MB. Copyright © 2018 Schwessinger et al. 2018 Schwessinger et al. This content is distributed under the terms of the Creative Commons Attribution 4.0 International license.

The observation that candidate effectors and BUSCOs show similar localization patterns relative to all genes and TEs led us to investigate if these two gene groups are spatially associated and if each group clusters with itself. We first compared the minimum distance between genes of the same group when subsampling to an equal number of genes in each group. Indeed, when we compared the minimum distances between candidate effectors, we found that these were less than the minimum distances between a random subset of genes (Fig. 5C). BUSCOs were also more closely associated with each other than a random subset of genes. Consistently, when we investigated the number of candidate effectors that clustered within a minimum given distance, we found that they were more clustered than BUSCOs or an equal-sized random subset of all genes (Fig. S6). A similar trend was observed, although to a lesser degree, for BUSCOs. Clustering of candidate effectors was also identified as a feature of several smut fungi, including *Ustilago maydis* and *Sporisorium scitamineum* (3, 76). In these related basidomycete plant pathogens, candidate effector gene clusters are born via tandem duplication, and linked TEs are hypothesized to contribute to the rapid evolution of these genes.

The observed spatial association of both BUSCOs and candidate effectors with themselves led us to investigate if these two gene groups are spatially associated with each other. Indeed, candidate effectors were located more closely with BUSCOs and vice versa than was a random subsample of all genes (Fig. 5D). This was a surprising observation, because BUSCOs are defined by their overall conservation, while candidate effectors are far less conserved. In obligate biotrophic fungi, a subset of effectors may be essential, because host colonization is an absolute requirement for survival. Therefore, there may be selection pressure on obligate biotrophs to favor recombination events that link some essential effectors to other essential genes (e.g., BUSCOs) to ensure their inheritance and conservation within the species complex. This is in contrast to plant pathogens that are also able to grow saprophytically, such as *Zymoseptoria tritici*, *V. dahliae*, *U. maydis*, and *Phytophthora infestans* (3, 75, 77, 78). In addition, the genetic variation within *P. striiformis* f. sp. *tritici* isolates in its center of genetic diversity is high, and sexual recombination may generate diverse effector complements that allow colonization of taxonomically distinct hosts, including barberry and grasses. In these natural environments, the composition of effector complements may be selectively neutral, and these processes may not facilitate effector gene compartmentalization. Once *P. striiformis* f. sp. *tritici* leaves the Himalayan region and invades large wheat-growing areas, sexual recombination is absent and hence effector gene compartmentalization is not possible.

### The candidate effector allele status influences association with conserved genes and evolutionary conservation.

We next investigated if the distance between candidate effectors and BUSCOs is correlated with their allelic variation. We calculated the normalized Levenshtein distance of cDNA and amino acid alignments for all allele pairs. The normalized Levenshtein distance measures the required single-character edits (insertions, deletions, or substitutions) to convert two strings into each other, e.g., an alignment of two allele sequences, while accounting for differences in sequence length. It can therefore be used as a proxy for sequence variation between two alleles (79). We did not observe any significant difference between the Levenshtein distances at the cDNA level when we compared BUSCOs and candidate effectors, whereas alleles of all other genes were more variable than candidate effectors (Table 2). This was in contrast to the variation seen at the protein level, where candidate effectors were more variable than BUSCOs (Table 2). This suggests that for candidate effectors, changes at the DNA level are more likely to result in changes to the protein sequence. We therefore also calculated the ratio of nonsynonymous to synonymous mutations for all alleles (dN/dS ratio) wherever possible (80). Indeed, analysis of the dN/dS ratios supported our previous observation that for candidate effectors, changes in the DNA sequence were more likely to alter the protein sequence (Table 2). This suggests that candidate effectors evolve faster than BUSCOs and most other allele pairs even though they are spatially associated with BUSCOs. The sequence variation in candidate effector allele pairs was not correlated with distance to the closest BUSCO, based on either Levenshtein distances on the protein level or dN/dS as a proxy (Spearman correlation, <|0.06|; *P* > 0.15). Subsequently, we investigated if candidate effector singletons were more distant from BUSCOs than their paired-allele counterparts. These singletons have either diverged dramatically from their ancestral allele counterparts, were lost due to structural rearrangements and mutations, or encode *de novo*-evolved candidate effectors. The candidate effector singletons were found to be located more distantly from BUSCOs than paired-allele candidate effectors (Fig. 6A) but were not more distant from other genes in general (Fig. 6B). Nonetheless, we reasoned that these candidate effector singletons might be more likely to be isolate or species specific, given their distinct genomic locations compared to paired-allele candidate effectors. We tested if candidate effector singletons are more likely to lack orthologs in publicly available *P. striiformis* f. sp. *tritici* genomes or other genomes of *Pucciniales* species (81). Out of a total of 453 candidate effector singletons, 116 lacked an ortholog in five other *P. striiformis* f. sp. *tritici* genomes, compared to 118 out of 1,272 allelic candidate effectors. Singletons are therefore more likely to be isolate specific than are paired-allele candidate effectors (Fischer’s exact test, *P* ≈ 1.36e^−16^). We observed a similar trend when we compared *Pst*-104E with the six publically available *Pucciniales* genomes. Of 985 candidate effectors lacking orthologs in other rust fungi, 313 were singletons and 672 allelic, also showing an enrichment for candidate effector singletons (Fischer’s exact test, *P* ≈ 4.45e^−26^).

**TABLE 2:** Candidate effector alleles are more variable than BUSCO alleles on the protein level

Comparison and parameter^a^	BUSCOs	Candidate effectors	Other genes
No. of loci with Levenshtein distance CDS	1,198	1,214	8,509
% of genes showing variation^b^	81	66	79
Median	0.0069	0.0044	0.0074
Mean	0.0288	0.0409	0.0579
Wilcoxon rank-sum test vs candidate effectors^c^	~9.47e−02	NA	~1.21e−10
			
No. of loci with Levenshtein distance protein	1,198	1,214	8,509
% of proteins showing variation^b^	65	60	70
Median	0.0028	0.0060	0.0075
Mean	0.0264	0.0474	0.0637
Wilcoxon rank-sum test vs candidate effectors^c^	~9.46e−05	NA^f^	~1.86e−10
			
No. of loci with dN/dS ratio^d^	859	619	5,403
% of loci showing variation^e^	75	87	85
Median	0.0802	0.3972	0.2840
Mean	0.2012	0.4797	0.3432
Wilcoxon rank-sum test vs candidate effectors^c^	~2.42e−54	NA	~1.91e−06

a Summary of normalized Levenshtein distances and dN/dS ratios calculated for CDS alignments and codon-based amino acid sequence alignments.

b Percentage of genes or proteins for which the normalized Levenshtein distance is >0.

c Calculated using the Wilcoxon rank-sum test with correction for multiple testing (Bonferroni; α = 0.05).

d Number of loci for which dN/dS ratios could be calculated using yn (Yang and Nielsen [80]).

e Percentage of loci for which dN/dS was not 0.

f NA, not applicable.

### Conclusions.

Using long-read sequencing technology, we are now starting to uncover the genomic diversity of dikaryotic fungi that was previously hidden by a reliance on short-read sequence assemblies. We used this approach to generate a highly contiguous haplotype-phased assembly of the Australian founder *P. striiformis* f. sp. *tritici* pathotype. We are now able to describe the levels of interhaplotype diversity, on both the structural and gene levels. It is difficult to fully evaluate the significance of observed levels of variations without additional experiments and in the absence of similar studies. With over 6.4% variation, the interhaplotype diversity of *Pst*-104E is higher than that reported for *P. coronata* f. sp. *avenae*, which ranges between 2.1 and 2.7% (74). It is also higher than the variation observed between two isolates of *Z. tritici* (isolates 3D7 versus MG2, 4.9%), an ascomycete pathogen of wheat that undergoes frequent sexual cycles (57, 75), and two isolates of *V. dahliae* (JR2 versus VdLs17, 1.7%), an ascomycete pathogen of tomatoes that propagates almost exclusively asexually (72). These comparisons suggest that the observed interhaplotype diversity of *P. striiformis* f. sp. *tritici* is high. *Pst*-104E belongs to the “North Western European” (NW European) lineage of *P. striiformis* f. sp. *tritici*, which has undergone long-term asexual reproduction. The NW European *P. striiformis* f. sp. *tritici* lineage can be traced back to its first sampling in the mid-1950s in the Netherlands, and it has not shown any signs of sexual recombination since (20, 82, 83). Consistent with this, two *P. coronata* f. sp. *avenae* isolates that showed much less interhaplotype variation than *Pst*-104E were from populations that reproduce both sexually and asexually on common buckthorn and oat, respectively (74). Frequent sexual recombination is likely to reduce interhaplotype diversity and to purge mutations that are deleterious in the monokaryon stage (84). On the other hand, long-term clonal lineages might accumulate polymorphisms that clear unwanted *Avr* genes but also contribute to genomic decay. It has long been hypothesized that prolonged clonal reproduction in the absence of sexual recombination and chromosomal reassortment will lead to high levels of heterozygosity between chromosomes that were initially homologous, a phenomenon known as the Meselson effect (85). This also suggests that *P. striiformis* f. sp. *tritici* isolates from the center of genetic diversity may display less interhaplotype diversity and a reduced allelic variation due to sexual recombination. This is an aspect of *P. striiformis* f. sp. *tritici* biology that we are aiming to test in future studies. With respect to this, it would be interesting to determine whether *Pst*-104E is still viable as a monokaryon in the absence of selection to retain gene function related to infection of barberry. The accumulation of large-scale polymorphisms and potentially deleterious mutations in each haploid genome of *Pst*-104E might have been buffered in the dikaryon stage, but it is likely that it represents a terminal lineage of *P. striiformis* f. sp. *tritici*, in agreement with Muller’s rachet hypothesis (84). Isolates from the NW European lineage show a reduction in teliospore production on wheat, the entry point into the *P. striiformis* f. sp. *tritici* sexual cycle, compared to isolates from the Himalayan region where sexual reproduction is common (86). Also, successful sexual reproduction under laboratory conditions has been reported only for *P. striiformis* f. sp. *tritici* isolates that emerged recently from the center of diversity in the Himalayan region (87), but not for isolates that have undergone long term clonal reproduction such as the NW European lineage (personal communication J. Rodriguez-Algaba). Lastly, *P. striiformis* f. sp. *tritici* populations of the NW European lineage have been completely replaced by more recent *P. striiformis* f. sp. *tritici* incursions in Europe and Australia (14, 24).

In the future, it will be important to generate high-quality genomes for more *P. striiformis* f. sp. *tritici* isolates, including from sexual populations in the Himalayan regions (88). This will enable us to understand the roles of sexual and asexual reproduction in the genome evolution of a dikaryon in the wild versus agricultural settings. For now, the near-complete haplotype-phased genome of *Pst*-104E provides a first haplotype-aware insight into the genetic architecture of a dikaryotic rust fungus that is pathogenic on wheat. In itself, it is a high-quality reference genome enabling investigation of the rapid and devastating evolution of the fungus to virulence during its asexual reproduction cycle in all wheat-growing areas today.

## MATERIALS AND METHODS

### *Puccinia striiformis* f. sp. *tritic*i pathotype, growth conditions, and spore amplification.

The isolate of pathotype 104E137A- was collected from the field in 1982 (Plant Breeding Institute accession number 821559=415), tested, and propagated as described previously (26). This pathotype is virulent on Heines VII (*Yr2*, *Yr25*), Vilmorin 23 (*Yr3*), hybrid 46 (*Yr4*), and Stubes Dickkopf, Nord Deprez, Suwon92/Omar & Avocet S (26). The rust propagated for PacBio sequencing was produced by selecting a single pustule of the original isolate (increase 0415Ga) on wheat plants of the susceptible variety Morocco. The initial inoculation involved rubbing leaves of the susceptible host with spores from a sterile cotton tip. Plants were incubated under plastic in the dark at 9.5°C for 18 h before being transferred to a greenhouse microclimate set at 22°C ± 2°C. After 6 days, plants were observed and all leaves were removed except for one leaf which showed signs of infection by a single fleck indicating that rust pustule was soon to erupt from the location. After pustule eruption, the single pustule selection was repeated to ensure that the starter material for propagation was a single genotype. Multiplication of rust was performed on *Triticum aestivum* cv. Morocco. For multiplication, 20 seeds of cultivar Morocco were placed as a single layer into 4-inch pots filled with pasteurized soil and watered with a half-strength solution of liquid fertilizer (Aquasol; Yates). At full coleoptile emergence, each pot was treated with 50 ml maleic hydrazide solution (2 ml liter^−1^ Slow Grow 270; Kendron). At full leaf emergence, plants were inoculated by rubbing with the pustules formed in the previous step and incubated as described previously. Once four pots of cultivar Morocco were heavily infected, spores were collected and inoculated onto 64 4-inch pots, and a differential set was established to check pathotype identity and purity. Rust spores were collected from the 64 pots by using a GRA-101 large-spore cyclone (Tallgrass Solutions) attached to a domestic vacuum cleaner. Spores were dried over silica gel for 7 days before being sieved through a 50-µm sieve and then stored at −80°C until DNA extraction.

### DNA extraction and genome sequencing.

DNA was extracted from dried dormant *P. striiformis* f. sp. *tritici* urediniospores as described in detail elsewhere (89). PacBio sequencing was performed at the Ramaciotti Centre (Sydney, Australia). For library preparation, the 20-kb BluePippin kit (PacBio) was used. DNA libraries were sequenced on a PacBio RSII instrument using P6-C4 chemistry. In total, we sequenced 13 SMRT cells (Table S1A). DNA samples from the same *P. striiformis* f. sp. *tritici* pathotype were also sequenced with Illumina short-read technology. We sequenced one TruSeq library on a HiSeq 2000 instrument as a 100-bp end library at the University of Western Sydney (Sydney, Australia). We sequenced one TruSeq PCR Free 250-bp end library on an Illumina MiSeq instrument at the Ramaciotti Center (Sydney, Australia).

### Genome assembly and manual curation.

For genome assembly, we used FALCON-Unzip github tag 1.7.4 with the parameters described in Files 12 and 13 at our study’s github repository (our study’s github page at https://github.com/BenjaminSchwessinger/Pst_104_E137_A-_genome) (29). We checked the resulting contigs for eukaryotic contamination by blastn searches against the NCBI nucleotide reference database (downloaded 4 May 2016) (37). None of the contigs had predominant noneukaryotic sequences as best BLAST hits at any given position. We performed two manual curation steps. In the first step, we reasoned that some of the primary contigs without haplotigs may actually represent haplotigs that could not be connected to their respective primary contigs in the assembly graph because there was too large a difference between the two haplotypes. We aligned all primary contigs without haplotigs to primary contigs with haplotigs by using mummer version 3 (34). We screened the best alignments of each primary contig without a haplotig for percentage alignment, length of alignments, and whether they aligned to regions in the primary contigs that previously had not been covered by a haplotig alignment. By using this approach, we reassigned 55 primary contigs without haplotigs (~6 Mb) to haplotigs (Table S1H). In the second step of manual curation, we removed all contigs with a mean coverage of greater 2,000× based on Illumina short-read data. In total, we removed 18 primary contigs (~0.6 Mb) and 7 haplotigs (~0.2 Mb), of which most were mitochondrial contigs based on blastn analysis. The final assembly contained 156 primary contigs (~83 Mb) and 475 haplotigs (~73 Mb) (Table S1H).

### Coverage analysis and identification of unphased regions in primary contigs.

We aimed to assess the coverage within contigs and between contigs by mapping Illumina short-read data on primary contigs (p) and primary contigs and haplotigs (ph) at the same time. We reasoned that unphased regions of primary contigs should have about twice the coverage of phased regions when mapped against ph and similar coverage when mapping for p versus ph. We trimmed Illumina short reads by using Trimmomatic v0.35 (90) (with the settings Illuminaclip:adapter.fa, 2:30:10; leading, 3; trailing, 3; slidingwindow, 4:25; minlen, 35) and assessed read quality with FastQC v0.11.4 (91). Reads were mapped against primary contigs only or against primary contigs and haplotigs by using BWA-MEM v0.7.15-r1142-dirty and the standard parameters (92). The coverage for each position was calculated with samtools v1.3.1 and a depth of the “-aa” flag (93). Unphased regions on primary contigs were defined as outlined above and converted to bed format. See the jupyter notebook Pst_104E_v12_coverage_analysis_ submission_21092017 in our github information.

We also performed a detailed coverage sequence depth analysis on 1-kb sliding windows by using 200-base intervals. We generated corresponding bed files with the window function in pybedtools for primary contigs and haplotigs. In addition, we generated corresponding sliding window bed files for primary contig regions that aligned with haplotig regions and for regions that lacked an associated haplotig. For this purpose, we combined initial sliding window bed files (see above) with .gff files illustrating the primary contig region that aligned with haplotigs (94, 95). The later .gff files were based on Assemblytics alignments of haplotigs to their respective primary contigs, determined by using nucmer (33). These bed files were used to calculate the mean base sequence depth, based on the samtools function bedcov (93). For details on how we generated the Assemblytics-based .gff file, see Pst_104E_v12_defining_alleles submission_21092017.ipynb. For details on this part of the coverage analysis, see the Revision_coverage_analysis.ipynb file in our gihub repository.

### Repeat annotation.

Repeat regions of the primary contigs and haplotigs were predicted independently. We used the REPET pipeline v2.5 (35, 96) for repeat annotation, in combination with Repbase v21.05 (45). First, we used TEdenovo to predict novel repetitive elements following the developer’s instructions and the parameters given in our github File 14. The set of TEs provided by TEdenovo were used to annotate all repetitive elements by using TEanno following the developer’s instructions, including the methodological advice, and the parameters given in File 15. Annotation was performed on genome version 0.4 and subsequently filtered for version 1.0 (Table S1G). We transferred the superfamily annotation according to the methods described by Wicker (36) for all elements from the underlying database hits if these agreed with each other and the REPET annotation. See jupyter notebooks Pst_104E_v12_TE_filtering_and_summary_p_contigs submission_21092017 and Pst_104E_v12_TE_filtering_and_summary_h_contigs submission_21092017 in our github repository for full analysis details.

### Estimation of TE age.

We estimated TE age based on the divergence of each sequence from the consensus sequence (38). We calculated the mean percent identity for all identified TEs (repbase2005_aaSeq, repbase2005_ntSeq, and *de novo*-identified repeats via TEdenovo) using the REPET pipeline function PostAnalyzeTELib.py -a 3 (File 1). We used the function *T* = *D*/*t* to roughly approximate TE age, where *T* is the elapsed time since the ancestral sequence, *D* is the estimated divergence based on percent identity calculated via the REPET pipeline [*D* = (1 − mean percent identity)/100], and *t* is the substitution rate per site per year. We estimated *t* to be ~2 × 10^−9^, based on previous publications (97, 98). For details, see the notebook Revision_TE_filtering_and_summary_p_contigs.ipynb at our study’s github respository.

### Gene model annotation.

We annotated genes on primary contigs and haplotigs independently. We combined RNA-seq-guided *ab initio* predictions by using CodingQuarry v2.0 (41) and BRAKER v1.9 (42) with *de novo* transcriptome assembly approaches of Trinity v2.2.0 (99) and PASA v2.0.1 (40). Gene models were unified using EvidenceModeler v1.1.1 (40) and the weights reported in File 16.

We mapped the trimmed RNA-seq reads described in this study (see below) and previously (39) against primary contigs and haplotigs by using hisat2 v2.1.0 (-max-intronlen 10000 -min-intronlen 20 -dta-cufflinks) (44). For *ab initio* predictions, we reconstructed transcripts using stringtie v1.2.3 (-f 0.2) (100). We ran CodingQuarry (-d) in the pathogen mode in SignalP4 (101) for secretome predictions on the soft-masked genome by using RepeatMasker v4.0.5 (-xsmall -s -GC 43). Similarly, we used the stringtie-reconstructed transcripts as a training set for the *ab initio* prediction pipeline BRAKER 1 v1.9 (42) and used the nonrepeat masked genome as a reference.

We used Trinity v2.2.0 to obtain *P. striiformis* f. sp. *tritici* transcripts both in the *de novo* mode and in the genome-guided mode (99). Several RNA-seq samples contained host and pathogen RNA, as they were prepared from infected wheat tissue. We first mapped all reads to primary contigs and haplotigs by using hisat2 (see above). We extracted mapped RNA-seq reads by using Piccard tools SamToFastq. Only the reads mapping against *P. striiformis* f. sp. *tritici* contigs were used in the *de novo* pipeline of Trinity (-seqType fq). For genome-guided assembly, we used bam files generated with hisat2 as the starting point for Trinity (-jacard_clip, -genome_gudied_max_intron 10000). We used the PASA pipeline v2.0.2 to align both sets of Trinity transcripts against *P. striiformis* f. sp. *tritici* contigs with BLAT and GMAP and the parameters given in File 17 (40).

The different gene models were combined using EvidenceModeler v.1.1.1 to get the initial gene sets for primary contigs and haplotigs (40). These were filtered for homology with proteins encoded in transposable elements. We used blastp to search for homology in the Repbase v21.07 peptides database, with an e value cutoff of 1e^−10^. In addition, we used transposonPSI to filter out genes related to TE translocation (46). We used the outer union of both approaches to remove genes coding for proteins associated with transposable elements from our list of gene models.

### Protein annotation.

For initial protein annotation, we used the fungus-centric annotation pipeline funannotate v0.3.10. This included annotations for proteins with homology to those reported in the databases swissprot (uniref90; downloaded 22 September 2016) (49), to carbohydrate-active enzyme (dbCAN, downloaded 22/9/2016) (48), to peptidases (Merops v10.0) (51), for proteins with eggnog terms (eggnog v4.5) (102), and SignalP4 (101). This annotation was complemented by using InterProScan v5.21-60 (-iprlookup -goterms -pa) (47), eggnog-mapper v0.99.2 (-m diamond and -d euk) (50), SignalP 3 (103), and EffectorP v1.01 (63, 104).

### Biological material and molecular biology methods for *P. striiformis* f. sp. *tritici* gene expression analysis.

We investigated *P. striiformis* f. sp. *tritici* gene expression in five different developmental stages or tissue types. We extracted total RNA from dormant spores, germinated spores after 16 h and 6 and 9 dpi of wheat, and from haustoria isolated from wheat leaves at 9 dpi.

In the case of dormant spores, spores were harvested from infected wheat at 14 to 18 dpi, dried under vacuum for 1 h, and stored at −80°C until use. For germination, fresh spores were heat treated for 5 min at 42°C and sprinkled on top of sterile Milli-Q (MQ) water. The container was covered with Clingfilm, and spores were incubated at 100% humidity at 10°C in the dark for 16 h before harvest. For infection assays, dormant spores were heat treated for 5 min at 42°C, mixed with talcum powder (1:7 [wt/wt]), and sprayed homogenously with a manual air pump onto 7-day-old wheat seedlings wetted with water by using a spray bottle. Plants were maintained in a container at 100% humidity in the dark at 10°C for 24 h. At this point, plants were transferred to a constant temperature growth cabinet at 17°C with a 16:8 light cycle. We collected infected wheat leaf samples 6 and 9 dpi. Haustoria were purified from wheat leaves at 9 dpi (105). Infected wheat leaves (~20 g) were surface sterilized with 70% ethanol, washed, and blended in 250 ml of 1× isolation buffer (1× IB; 0.2 M sucrose, 20 mM morpholinepropanesulfonic acid [pH 7.2]). The homogenate was passed consecutively through 100-µm and 20-µm meshes to remove cell debris. The filtrate was centrifuged at 1,080 × *g* for 15 min at 4°C, and the resulting pellets were resuspended in 80 ml 1× IB containing 30% (vol/vol) Percoll. The suspension was centrifuged at 25,000 × *g* for 30 min at 4°C. The upper 10 ml of each tube was recovered, diluted 10 times with 1× IB, and centrifuged at 1,080 × *g* for 15 min at 4°C. The pellets were resuspended in 20 ml of 1× IB containing 25% (vol/vol) Percoll and centrifuged at 25,000 × *g* for 30 min at 4°C. The upper 10 ml of each tube was recovered, diluted 10 times in 1× IB, and centrifuged at 1,080 × *g* for 15 min at 4°C. Pellets were stained with concanavalin A-Alexa Fluor 488 to visualize haustoria under a fluorescence microscope. The final pellets were frozen in liquid nitrogen and stored at −80°C prior to RNA isolation.

RNA for all samples was isolated as follows. Total RNA was isolated using the Qiagen plant RNeasy kit following the manufacturer’s instructions. Initial RNA quality and purity checks were performed on a NanoDrop ND-1000 UV-vis spectrophotometer. Samples were treated with DNase I (New England Biolabs), following the manufacturer’s instructions. Samples were purified using the Qiagen plant RNeasy kit following the cleanup protocol, and RNA was eluted from columns in 50 μl of RNase-free water. The concentration and integrity of all final RNA samples were verified on the Agilent 2100 bioanalyzer, using the RNA 6000 nano and pico kits. Three biological replicates were processed.

RNA samples were sequenced at the Ramaciotti Centre (Sydney, Australia) on an Illumina HiSeq 2000 instrument as 100-bp paired-end reads. Approximately 10 μg of total RNA per biological sample was processed with the TruSeq RNA sample preparation kit v2.

### Differential expression analysis.

We trimmed Illumina RNA-seq reads by using Trimmomatic v0.35 (90) (parameters of Illuminaclip:adapter.fa, 2:30:10; leading, 3; trailing, 3; slidingwindow, 4:25; minlen, 35), and we assessed read quality with FastQC v0.11.4 (91). We mapped reads using gene models as a guide and STAR v020201 (106). We first generated a genome reference in the genomeGenerate mode using our .gff for gene models (-runMode genomeGenerate –sjdbGTFfile -sjdbGTFtagExonParentTranscript Parent). We mapped our RNA-seq reads against this reference by using STAR in the alignReads mode (-runMode alignReads readFilesCommand gunzip –c outFilterType BySJout –outFilterMultimapNmax 20 -alignSJoverhangMin 8 -alignSJDBoverhangMin 1, -outFilterMismatchNmax 999 -alignIntronMin 20 -alignIntronMax 10000 -alignMatesGapMax 1000000 -outSAMtype BAM SortedByCoordinate -outSAMstrandField intronMotif -outFilterIntronMotifs RemoveNoncanonical -quantMode GeneCounts). We used featureCounts v1.5.3 and our gene annotation to quantify the overlaps of mapped reads with each gene model (-t exon -g Parent) (107). We identified differentially expressed genes in either haustoria or infected leaves relative to expression levels in germinated spores (|log fold change|, >1.5; adjusted *P* < 0.1) using the DESeq2 R package (108). *k*-means clustering was performed on average rlog-transformed values for each gene and condition. The optimal number of clusters was defined by using the elbow plot method and circular heat maps drawn using Circos (109). Scripts regarding the gene expression analysis can be found in the gene_expression folder of the github repository.

We compared the expression pattern of alleles in different clusters (Table S1F and G) in jupyter notebook Pst_104E_v12_secretome_expression_cluster_analysis submission_21092017 in the github repository.

### BUSCO analysis.

We used BUSCO2 v2.0 4 beta to identify core conserved genes and to assess genome completeness (31). In all cases, we ran BUSCO2 in the protein mode, using the *Basidiomycota* reference database downloaded 9 January 2016 (-l basidiomycota_odb9 -m protein). We combined BUSCO identification on primary contigs and haplotigs nonredundantly to asses completeness of the combined assembly. For details, see jupyter notebook Pst_104E_v12_BUSCO_summary submission_21092017 in the github repository.

### Interhaplotype variation analysis.

We mapped trimmed reads against primary contigs using BWA-MEM v0.7.15-r1142-dirty with the standard parameters (92). We called SNPs with FreeBayes default parameters (110) and filtered the output with vcffilter v1.0.0-rc1 (-f “DP >10” -f “QUAL >20”) (111). SNP calls were summarized by using real-time genomic vcfstats v3.8.4 (112).

We aligned all haplotigs to their corresponding primary contigs by using nucmer of the mummer package (-maxmatch -l 100 -c 500) (34). We fed these alignments into Assemblytics to estimate the interhaplotype variation for each primary contig-haplotig pairing (33). For this analysis, we used a unique anchor length of 8 kb, based on the length of identified TEs in our *P. striiformis* f. sp. *tritici* assembly and a maximum feature length of 10 kb. For consistency, we used nucmer alignments filtered by Assemblytics for the allele status analysis (see below). Analysis and summary of variations is shown in jupyter notebook Pst_104E_v12_assemblytics_analysis submission_2109 2017 and Pst_104E_v12_nucmer_and_assemblytics submission_21092017 in the github repository.

### Allele status analysis.

We used proteinortho v5.16 in synteny mode with default parameters (-synteny) to identify alleles between the primary assembly and haplotigs (52). We parsed the results and defined three major allele status categories, as follows. Allele pairs were parsed from the poff-graph output file. Interhaploid genome paralogs were parsed from the proteinortho output file and checked for absence in the poff-graph output file. Potential singletons were defined as gene models that were absent from both of these two output files. Alleles were further subdivided into alleles for which the primary and associated haplotig gene models were located on contigs that aligned with each other at the position of the primary gene model (Fig. S2A), alleles for which the primary and associated haplotig gene models were located on contigs that did not align with each other at the position of the primary gene model (Fig. S2B), and alleles for which the allele of a primary gene model was not located on a haplotig associated with the respective primary contig (Fig. S2C). Potential singletons were screened for being located in regions of the primary assembly that were unphased based on Illumina coverage analysis (see above). Genes located in these regions were defined as unphased and removed from the initial list. All other gene models constitute haplotype-specific singletons. Analysis details can be found in the jupyter notebooks Pst_104E_v12_defining_alleles submission_21092017 and Pst_104E_v12_missing_allele_QC submission_21092017.

### Allele variation analysis.

We assessed the variation of allele pairs by using three approaches. We calculated the Levenshtein distance (79) on the CDS alignments of two alleles on the codon-based protein alignments, and we calculated the dN/dS ratios by using these two alignment sets with yn00 paml version 4.9 (80). The CDS of two alleles were aligned using muscle v3.8.31 (113), and codon-based alignments were generated using PAL2NAL v14 (114). The Levenshtein distance was calculated in python using the distance module v0.1.3. Analysis details can be found in jupyter notebook Pst_104E_v12_post_allele_analysis submission_21092017.

### Genome architecture analysis.

We used bedtools v2.25.0 (94) and the python module pybedtools (95) to perform various genome analysis tasks. This included the calculation of nearest neighbors using the closest function. Details of the analysis can be found in jupyter notebooks Pst_104E_v12_post_allele_analysis submission_21092017 and Pst_104E_v12_effectors submission_21092017.

### Orthology analysis of candidate effector analysis.

We performed orthology analysis with proteinortho v5.16 (-singles) (52) of all nonredundant candidate effectors with publicly available *P. striiformis* f. sp. *tritici* genomes. *Pst*-130 (4) and *Pst*-78 (28) protein sets were downloaded from MycoCosm (9 May 2017) (81). *Pst*-0821, *Pst*-21, *Pst*-43, and *Pst*-887 were downloaded from yellowrust.com (30 March 2017) (5). We performed a similar analysis to search for candidate effector orthologs in *Pucciniales* excluding *P. striiformis* f. sp. *tritici* genomes. *Puccinia triticina* 1-1 BBBD Race 1 (28), *Puccinia graminis* f. sp. *tritici* v2.0 (6), *Puccinia coronata* f. sp. *avenae* isolates 12SD80 and 12NC29 (74), and *Melampsora lini* CH5 (115) genomes were downloaded from MycoCosm (9 May 2017). The *Puccinia sorghi* genome (116) (ASM126337v1) was downloaded from NCBI (9 May 2017).

### Data and statistical analysis.

We used the python programing language (117) in the jupyter notebook environment for data analysis (118). In particular, we used pandas (119), numpy (120), matplotlib (121), and seaborn for data processing and plotting. Statistical analysis was performed using the Scipy (120) and statsmodel toolkits.

### Data availability.

The data generated in the course of this study, which is registered as Bioproject number PRJNA396589, were assigned NCBI accession numbers as follows: short read archive accession numbers SRX311905 and SRX311918 to SRX311920 for the PacBio 10- to 20-kb BluePippin kit, RSII, and 13 SMRT cells; SRX311916 and SRX311917 for the genomic DNA TruSeq library of the HiSeq 2000 100-bp paired-end library; SRX311915 for the genomic DNA TruSeq PCR-free MiSeq, 250-bp paired-end library; and SRX3191029 to SRX3191043 for the TruSeq v2 RNA-seq samples and HiSeq 2000 100-bp paired-end library.

Bioinformatic scripts, additional supplemental data files, and genome annotations can be found on our manuscript’s github page, https://github.com/BenjaminSchwessinger/Pst_104_E137_A-_genome. The genome is also available with MycoCosm (https://genome.jgi.doe.gov/Pucstr1/Pucstr1.home.html).
