# Supplementary material for: A Near-Complete Haplotype-Phased Genome of the Dikaryotic Wheat Stripe Rust Fungus Puccinia striiformis f. sp. tritici Reveals High Interhaplotype Diversity
Source: mBio. 2018 Feb 20;9(1):e02275-17. doi: 10.1128/mBio.02275-17 (PMC5821087; doi:10.1128/mBio.02275-17)
Supplement: TABLE S1 [file mbo001183717st1.docx]

**Supplemental Tables:**

|  | bases |
| --- | --- |
| Total sequence amount | 13,702,106,090 |
| Number of reads | 1,279,425 |
| Median read length | 9,652 |
| Mean read length | 10,710 |
| N75 read length | 9,984 |
| N50 read length | 15,196 |
| N25 read length | 19,673 |
| Maximum read length | 52,241 |

**Supplemental Table 1A: Summary table of PacBio genome sequencing using 13 SMRT cells**

|  | All genes [#/%] | | BUSCOs [#/%] | | Candidate effectors [#/%] | |
| --- | --- | --- | --- | --- | --- | --- |
|  | Primary contigs | Haplotigs | Primary contigs | Haplotigs | Primary contigs | Haplotigs |
| protein coding genes | 15928 | 14321 | 1444/9.07 | 1292/9.02 | 1572/9.70 | 1388/9.70 |
| mean length protein | 394 | 392 | 606 | 591 | 252 | 241 |
| GO terms | 5949/37.35 | 5406/37.75 | 1386/95.98 | 1241/96.05 | 145/9.22 | 116/8.36 |
| InterPro domain match | 6678/41.93 | 6068/42.37 | 1399/96.88 | 1249/96.67 | 193/12.28 | 165/11.89 |
| Pfam domain | 5950/37.36 | 5380/37.57 | 1352/93.63 | 1209/93.58 | 165/10.50 | 143/10.30 |
| EggNog term | 7679/48.21 | 6919/48.31 | 1433/99.24 | 1281/99.15 | 206/13.10 | 164/11.82 |
| KEGG Pathway | 2712/17.03 | 2434/17.00 | 811/56.16 | 727/56.27 | 43/2.74 | 32/2.31 |
| Merops domain | 245/1.54 | 236/1.65 | 14/0.97 | 11/0.85 | 31/1.97 | 35/2.52 |
| CAZy domain | 272/1.71 | 230/1.61 | 54/3.74 | 47/3.64 | 10/0.64 | 11/0.79 |
| SignalP3 | 2430/15.26 | 2207/15.41 | 27/1.87 | 27/2.09 | 1572/100.00 | 1388/100.00 |
| without domain | 7590/47.65 | 6803/47.50 | 2/0.14 | 1/0.08 | 1307/83.14 | 1173/84.51 |

**Supplemental Table 1B: Even annotation of primary contigs and haplotigs yet divergent annotation of BUSCOs and candidate effectors**

Annotation summary table of all protein coding genes using a combination of different annotation sources and pipelines. For each category the number of proteins and the percentage of proteins having a hit within the category is given.

First number in each column indicates the total number of proteins and the second number the percentage within each category.

|  | Primary contigs | Haplotigs | Number of pairs | Total number of genes |
| --- | --- | --- | --- | --- |
| All genes | 15928 | 14321 | N.A. | 30249 |
| **With an allele** | 10785 | 10860 | 10921 | 21645 |
| Allele on overlapping haplotig^A^ | 9627 | 9735 | 9756 | 19362 |
| Allele on non-overlapping associated haplotig^B^ | 443 | 450 | 450 | 893 |
| Allele on non-associated haplotig^C^ | 715 | 709 | 715 | 1424 |
| **Conserved in other haplotype but not syntenic** | 382 | 530 | N.A. | 912 |
| **Haplotype singleton** | 4098 (4761) | 2931 | N.A. | 7029 |
| Single Haplotype genes | 1346 | 160 | N.A. | 1506 |

**Supplemental Table 1C: Summary of inter-haplome allele analysis**

Summary table describing the allele and conservation state of *Pst*-104E genes. Primary contigs and haplotigs were treated as two representative units for orthology analysis in Proteinortho with the syntany flag. The three major categories are highlight in bold.

In case of alleles they can be subdivided in three categories as illustrated in Supplemental Figure 2. Superscript A, B, C, correspond to Supplemental Figure 2 A, B, C.

The number in brackets for haplotype singletons is the number of singletons given by Proteinortho without filtering for unphased gene models based on genome coverage analysis. True haplotype singletons are located in phased regions of the genome.

Single haplotype genes are haplotype singletons that do not have a significant blast hit when using the other genome assembly as reference (blastn, e-value < 0.01)

| Rust genome | # of proteins | # of proteins predicted as secreted |
| --- | --- | --- |
| *Pgt* 21-0 | 22,391 | 2,506 (11.19%) |
| *Pgt* | 15,979 | 2,066 (12.93%) |
|  |  |  |
| *Pst* 104 (primary) | 15,928 | 2,430 (15.26%) |
| *Pst* 104 (haplotigs) | 14,321 | 2,207 (15.41%) |
| *Pst* 21 | 20,487 | 2,152 (10.50%) |
| *Pst* 43 | 20,814 | 2,015 ( 9.68%) |
| *Pst* 887 | 20,396 | 1,417 ( 6.95%) |
| *Pst* 78 | 20,482 | 2,502 (12.22%) |
| *Pst* 0821 | 20,636 | 1,604 ( 7.77%) |
| *Pst* 130 | 18,021 | 1,944 (10.79%) |
|  |  |  |
| *Puccinia triticina* | 15,685 | 1,645 (10.49%) |
|  |  |  |
| *Pca* 12SD80 | 26,321 | 2,473 ( 9.40%) |
| *Pca* 12NC29 | 28,270 | 2,591 ( 9.17%) |

**Supplemental Table 1D: Number of total proteins and predicted proteins in publically available grass rust genomes**

Numbers in brackets indicates the percentage of protein that are predicted to be secreted in each proteome using SignalP3.

| Clusters | # proteins | average protein length (aas) | % EffectorP | % NLS (LOCALIZER 1.0.2) | % ApoplastP |
| --- | --- | --- | --- | --- | --- |
| 1 | 148 | 334 | 45.9% | 16.9% | 48% |
| 2 | 274 | 344 | 38% | 25.2% | 24.1% |
| 3 | 227 | 266 | 53.7% | 14.1% | 52.4% |
| 4 | 278 | 373 | 40.6% | 20.5% | 41% |
| 5 | 291 | 309 | 46.7% | 21.6% | 35.1% |
| 6 | 60 | 248 | 46.7% | 6.7% | 50% |
| 7 | 280 | 296 | 51.4% | 17.1% | 41.4% |
| 8 | 308 | 410 | 26% | 40.6% | 8.1% |

**Supplemental Table 1E: Summary table of genes located in indicated expression clusters in regards to EffectorP, nuclear localization and apoplastic localization prediction**

We used EffectorP, Localizer, and ApoplastP for predictions.

| Allele state | Cluster1 | Cluster2 | Cluster3 | Cluster4 | Cluster5 | Cluster6 | Cluster7 | Cluster8 |
| --- | --- | --- | --- | --- | --- | --- | --- | --- |
| allelic | 126 | 224 | 190 | 217 | 261 | 47 | 220 | 272 |
| non-allelic protein 'ortholog' | 0 | 3 | 2 | 6 | 3 | 0 | 3 | 5 |
| singleton | 22 | 47 | 35 | 55 | 27 | 13 | 57 | 31 |
| Total genes | 148 | 274 | 227 | 278 | 291 | 60 | 280 | 308 |
|  | percentages of haplotig alleles expressed in given cluster | | | | | | | |
| Cluster9 | 70.9 | 0.5 | 0 | 52.4 | 1.2 | 0 | 0.5 | 0 |
| Cluster10 | 0.9 | 28.2 | 2.2 | 0 | 59.7 | 0 | 28.8 | 0.8 |
| Cluster11 | 0 | 30.1 | 8.7 | 0 | 0.8 | 0 | 0.9 | 1.9 |
| Cluster12 | 18.8 | 0 | 0 | 0 | 0 | 91.1 | 0 | 0 |
| Cluster13 | 0 | 2.8 | 79.2 | 0 | 0 | 0 | 9.4 | 0.4 |
| Cluster14 | 0 | 27.8 | 3.3 | 0.5 | 0.4 | 0 | 0.5 | 34.6 |
| Cluster15 | 0 | 0.5 | 0 | 0 | 0.4 | 0 | 0 | 50.8 |
| Cluster16 | 0 | 0.5 | 0.5 | 31.2 | 22.6 | 0 | 44.8 | 0 |
| Total | 90.6 | 90.3 | 94 | 84.1 | 85.1 | 91.1 | 84.9 | 88.5 |

**Supplemental Table 1F: Summary of the allele state and orthologous expression pattern of secreted protein coding genes located on primary contigs**

The top half of the table shows how many genes are within each cluster and of those how many are allelic, non-allelic protein ‘orthologs’ or singletons.

The bottom half of the table shows how the expression of alleles of primary genes cluster in the haplotig gene expression analysis.

| Allele state | Cluster9 | Cluster10 | Cluster11 | Cluster12 | Cluster13 | Cluster14 | Cluster15 | Cluster16 |
| --- | --- | --- | --- | --- | --- | --- | --- | --- |
| allelic | 223 | 303 | 101 | 69 | 187 | 168 | 148 | 247 |
| non-allelic protein 'ortholog' | 8 | 7 | 4 | 4 | 7 | 7 | 0 | 5 |
| singleton | 17 | 22 | 10 | 6 | 11 | 22 | 8 | 30 |
| Total | 248 | 332 | 115 | 79 | 205 | 197 | 156 | 282 |
|  | percentages of primary alleles expressed in given cluster | | | | | | | |
| Cluster1 | 37.2 | 0.3 | 0.0 | 31.9 | 0.0 | 0.0 | 0.0 | 0.0 |
| Cluster2 | 0.4 | 20.1 | 64.4 | 0.0 | 3.2 | 35.7 | 0.7 | 0.4 |
| Cluster3 | 0.0 | 1.3 | 15.8 | 0.0 | 77.5 | 3.6 | 0.0 | 0.4 |
| Cluster4 | 48.9 | 0.0 | 0.0 | 0.0 | 0.0 | 0.6 | 0.0 | 26.3 |
| Cluster5 | 1.3 | 48.8 | 2.0 | 0.0 | 0.0 | 0.6 | 0.7 | 22.7 |
| Cluster6 | 0.0 | 0.0 | 0.0 | 59.4 | 0.0 | 0.0 | 0.0 | 0.0 |
| Cluster7 | 0.4 | 20.1 | 2.0 | 0.0 | 10.7 | 0.6 | 0.0 | 38.5 |
| Cluster8 | 0.0 | 0.7 | 5.0 | 0.0 | 0.5 | 53.6 | 89.2 | 0.0 |
| Total | 88.3 | 91.4 | 89.1 | 91.3 | 92.0 | 94.6 | 90.5 | 88.3 |

**Supplemental Table 1G: Summary of the allele state and orthologous expression pattern of secreted protein coding genes located on haplotigs**

The top half of the table shows how many genes are within each cluster and of those how many are allelic, non-allelic protein ‘orthologs’ or singletons.

The bottom half of the table shows how the expression of alleles of haplotig genes cluster in the primary gene expression analysis.

| Version |  | number of contigs | size [bases] | number of contigs | size [bases] |
| --- | --- | --- | --- | --- | --- |
| v0.1 | Falcon | 297 | 92345074 | 429 | 37495020 |
| v0.2 | Falcon-Unzip | 235 | 89868210 | 512 | 68673037 |
| v0.3 | Falcon-Unzip-Quiver | 229 | 89887834 | 427 | 67710241 |
| v0.4 | manual curration of primary contigs to haplotigs | 174 | 83950403 | 482 | 73647672 |
| v1.0 | removal of high coverage contigs e.g. repeats and mitochondria | 156 | 83355616 | 475 | 73478481 |

**Supplemental Table 1H: Changes in genome size and contig number at different steps in the assembly process**
