# Supplementary figures and images for: A Near-Complete Haplotype-Phased Genome of the Dikaryotic Wheat Stripe Rust Fungus Puccinia striiformis f. sp. tritici Reveals High Interhaplotype Diversity
Source: mBio. 2018 Feb 20;9(1):e02275-17. doi: 10.1128/mBio.02275-17 (PMC5821087; doi:10.1128/mBio.02275-17)

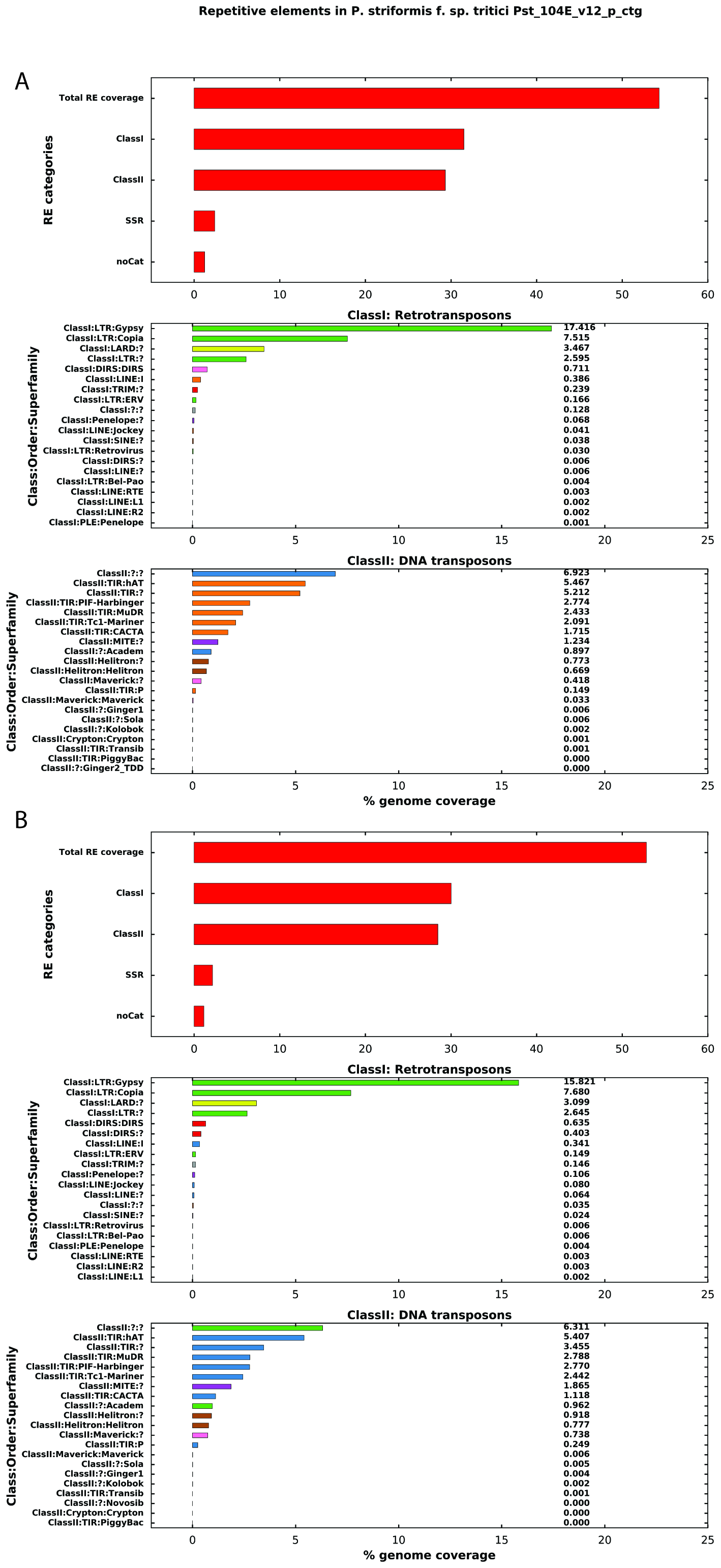

Supplement: FIG S2 [file mbo001183717sf2.tif]

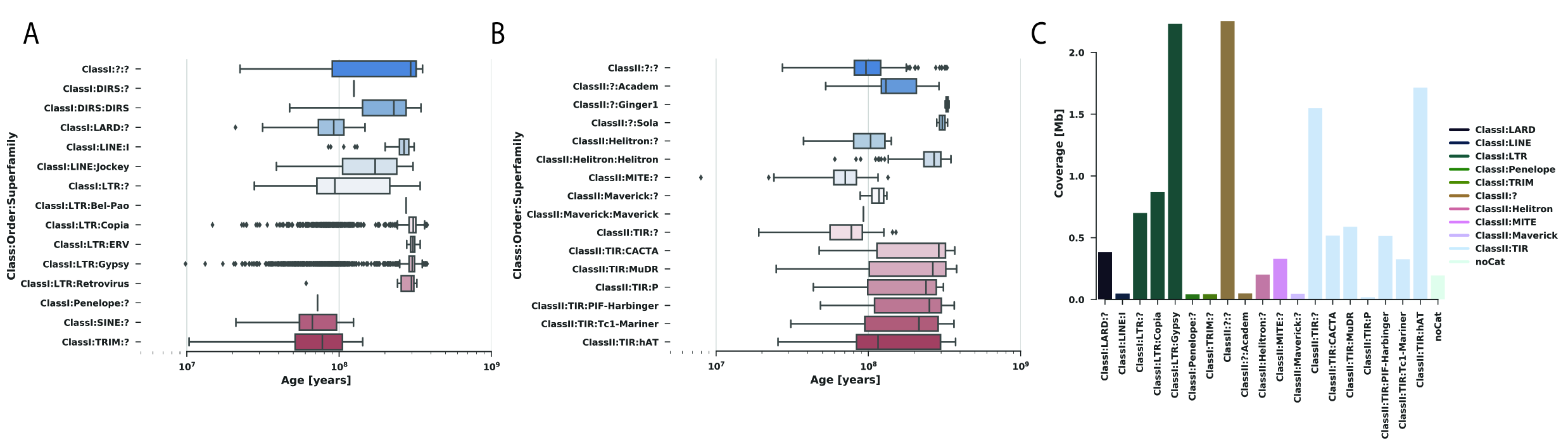

Supplement: FIG S3 [file mbo001183717sf3.tif]

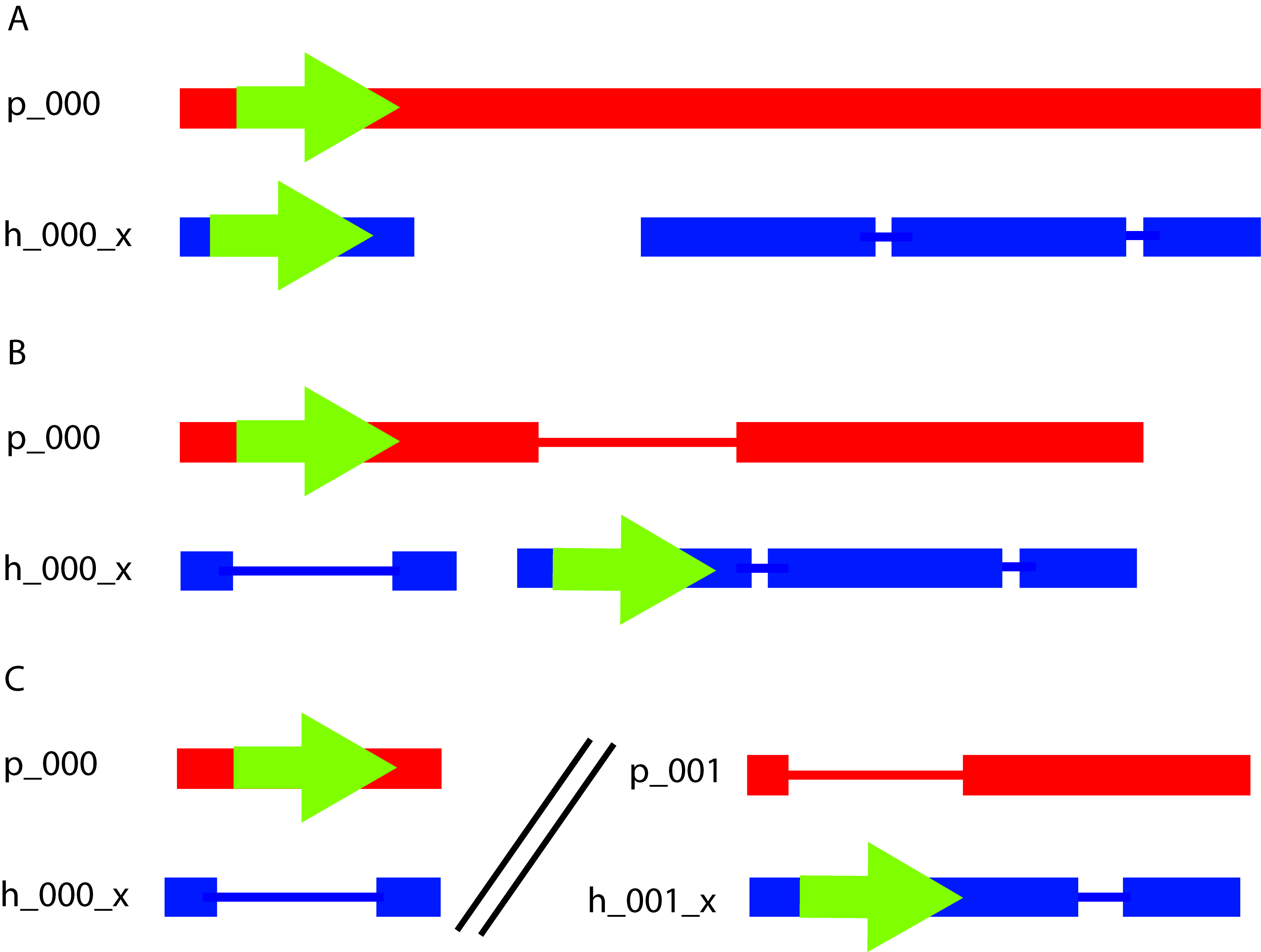

Supplement: FIG S4 [file mbo001183717sf4.tif]

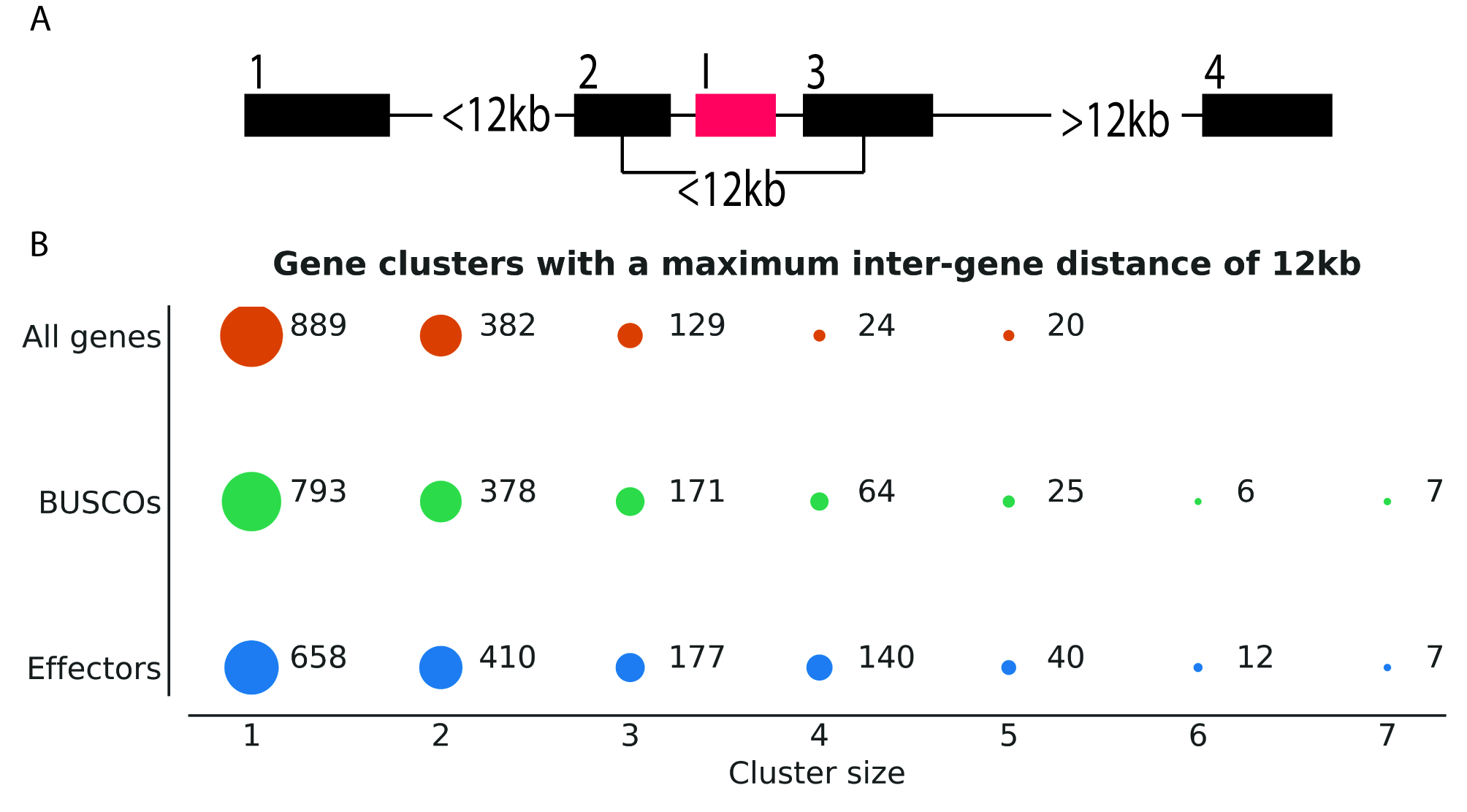

Supplement: FIG S6 [file mbo001183717sf6.tif]
